# Synthesis of Resins Using Epoxies and Humins as Building Blocks: A Mechanistic Study Based on In-Situ FT-IR and NMR Spectroscopies

**DOI:** 10.3390/molecules24224110

**Published:** 2019-11-14

**Authors:** Xavier Montané, Roxana Dinu, Alice Mija

**Affiliations:** Institut de Chimie de Nice, Université Côte d’Azur, Université Nice-Sophia Antipolis, UMR CNRS 7272, CEDEX 02, 06108 Nice, France; Xavier.Montane@unice.fr (X.M.); Roxana.CONDRUZ@unice.fr (R.D.)

**Keywords:** biomass, green chemistry, mechanims, humins, epoxy resins, thermosets

## Abstract

The combination of eco-respectful epoxy compounds with the humins, a by-product of biomass chemical conversion technologies, allow the obtention of materials with high added value. In this work, we propose a chemical connection study of humins with two aliphatic bis-epoxides through copolymerization reactions to synthesize sustainable, bio-based thermosets. The mechanism insights for the crosslinking between the epoxides and humins was proposed considering the different functionalities of the humins structure. Fourier Transform InfraRed (FT-IR), one dimensional (1D) and two-dimensional (2D) Nuclear Magnetic Resonance (NMR) spectroscopy techniques were used to build the proposed mechanism. By these techniques, the principal chain connections and the reactivity of all the components were highlighted in the synthesized networks.

## 1. Introduction

Nowadays, there is an increasing demand to produce renewable resource-based materials due to the rapid exhaustion of fossil fuels [1,2,3,4]. The design and synthesis of polymeric materials developed from renewable resources like vegetable oils or lignocellulosic biomass have successfully shown their potential as alternatives to the petroleum-based polymers [5,6]. A class of polymers that are widely used due to their variety of excellent properties including adhesion, mechanical performance, thermal resistance and chemical environmental stability are the epoxy resins. Nowadays, epoxy monomers can be obtained from plant oils or from other bio-based raw materials [7,8,9]. One of these examples is the glycerol diglycidyl ether (GDE), which is obtained from glycerol. Glycerol is a triol component of triglyceride vegetable oils. Moreover, glycerol can be obtained as a by-product in the synthesis of biodiesel via the transesterification of vegetable oils [10]. Poly(ethylene glycol) diglycidyl ether (PEGDE) is a further bis-epoxide that is used in a wide variety of applications, from industrial applications to medicine. PEGDE is obtained from ethylene glycol, a green raw material that can be obtained from starch [5].

On the other hand, the valorization of industrial waste has started to receive more attention owing to the required transition from a linear economy to a circular one. This transition is nowadays attracting a great deal of interest due to the high price and future depletion of fossil fuel stocks. One of the industrial wastes that has been used for the synthesis of polymers are humins [11,12]. Humins are carbonaceous, heterogeneous and polydisperse by-products obtained during the acid-catalyzed dehydration of sugars (ACD). In these processes, the formation of humins is still unavoidable. Humins chemical structure is based on a network of furanic rings linked via aliphatic chains bearing reactive oxygen-based functional groups (hydroxyls, acids, ketones, aldehydes, esters, …) [13,14,15]. The study of the formation and characterization of these side products are of great interest. In fact, different routes have been proposed for humins formation [16,17,18,19,20].

Humins demonstrate high potential in different applications during the recent years. They can be used as a source of sustainable H_2_ and as a model feedstock for gasification [21]. Besides, they can be used in the preparation of nanocomposites, sorbents and electrode materials [22,23,24]. Likewise, other polymeric materials were synthesized by using humins as raw materials. As an example, our group reported also the synthesis of humins-derived porous materials [25,26].

The combination of eco-friendly epoxy monomers with humins allows for the development of materials with high added value: The introduction of furanic structures into the polymeric networks will provide an improvement of the rigidity and give a high thermal stability to the resulting epoxy thermosets.

In this work, we propose the investigation of chemical connection of the humins as macromonomers with epoxide structures through copolymerization reactions to synthesize fully sustainable thermosets. Humins were combined with two epoxy monomers using different weight ratios. To promote their covalent interactions, a tertiary amine (*N,N*-Benzyldimethylamine, BDMA) was used as initiator. For the synthesis of the thermosets, neither chemical modifications nor solvent were needed, which agrees with the green chemistry principles. The mechanism insights for the crosslinking between the epoxy molecules and the humins was proposed considering the different functionalities of the compounds. The main starting hypothesis of this work is that the carboxylic acid groups and the hydroxyl groups of humins could open the epoxy rings of both glycerol digylcidyl ether (GDE) and poly(ethylene glycol) diglycidyl ether (PEGDE) [27,28,29,30]. However, different secondary reactions could theoretically occur: The catalyzed homopolymerization of epoxides, the formation of Diels–Alder adducts between different furan rings of humins, the nucleophilic attack of alkoxide anions to the carbonyl groups in humins, or the autopolycondensation of humins.

Recently, we reported on the thermomechanical properties of the humins/PEGDE, humins/GDE and humins/PEGDE/GDE copolymers [31]. The prepared humins copolymers have a ductile and elastomeric character, with a tensile strain at break reaching ≈ 60 % that is an excellent result for a polyfuranic network. Continuing the interesting results of our precedent study, this work focusses on the elucidation of the chemical pathway and reaction scenarios conducting at the synthesis of these new copolymers. The purpose is to determine the functionalities that lead to the chemical connections between humins and the epoxides and to highlight the principal reactivities by in situ Fourier Transform InfraRed (FT-IR) spectroscopy, 1D and 2D Nuclear Magnetic Resonance (NMR) spectroscopy.

## 2. Results

### Synthesis of Copolymers

Three different formulations were prepared. The composition of each formulation is summarized in Table 1. The composition of the three different formulations were studied to evaluate the effect of the two aliphatic epoxy monomers: GDE and PEGDE, when they were mixed with humins. The three proposed formulations contain a high amount of humins, the main objective being the valorization of this by-product. The reactivity of each bis-epoxide and the crosslinking reactions were studied. In addition, a third formulation that contains the same ratio of both bis-epoxides was prepared and analyzed.

## 3. Discussion

### 3.1. Plausible Polymerization Mechanism

The complexity of humins was studied by different authors. In fact, the combination of different techniques such FT-IR, pyrolysis GC-MS and liquid and solid-state NMR suggested a furan-rich structure containing several oxygen functionalities (carboxylic acids, aldehydes, ketones, hydroxyl groups, etc.), in which the furan rings are connected directly or via aliphatic linkages [18].

The presence of different functionalities in humins could lead to different reactivities in the presence of bis-epoxide comonomers. Nevertheless, one could expect that the predominant reaction in these systems is the ring-opening polymerization of diepoxy monomers. The initiation of the ring-opening polymerization of an epoxy compound using a tertiary amine as initiator (as in the case of BDMA in our study) requires the presence of a proton donor or electrophilic agent such as a carboxylic acid or hydroxyl groups [32].

**Scheme 1:**
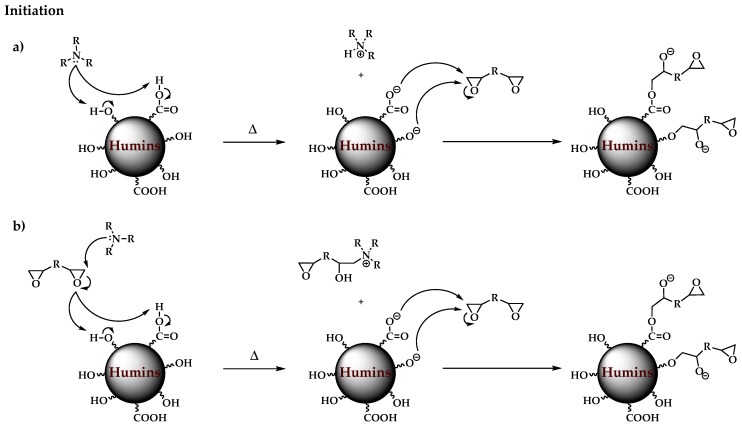

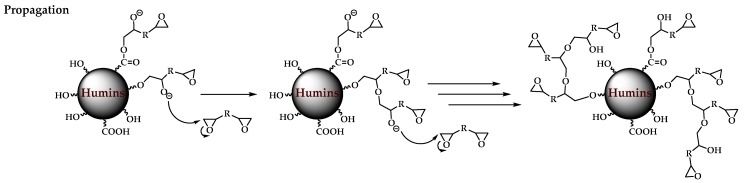
Proposed mechanism for the initiation and propagation of the ring-opening polymerization of the diepoxy monomers with humins using benzyldimethylamine (BDMA) as initiator.

The large number of hydroxyl groups that are present in humins combined with the carboxylic acid groups could initiate epoxy ring polymerization through a nucleophilic attack to the less-hindered carbon, as shown in Scheme 1. In the case of humins, the tertiary amine initiator helps to transfer the carboxylic acid and the hydroxyl to carboxylate and alkoxide anions respectively, which are the true nucleophiles that opens the epoxy rings (initiation step a). Furthermore, the hydroxyl and carboxylic acid groups can form a complex with the oxygen in the epoxy ring and the tertiary amine. This interaction increases the positive charge in the oxirane ring methylene carbons, which then undergoes via nucleophilic attack by the lone pair of electrons of the tertiary amine. In this manner, a carboxylate, an alkoxide and a mobile quaternary ammonium ion are formed between the oxirane and the amine (initiation step b). Then, the alkoxide formed is active to opening another epoxy ring and the reaction further proceeds until completion (propagation step) [33]. In the terpolymerization (formulation HP20G20B5), a mixture of poly(ethylene glycol) diglycidyl ether and glycerol diglycidyl ether in the same weight ratio were mixed with humins. Due to the presence of both epoxide structures, a random combination between both PEGDE and GDE epoxy monomers in the growing polymer chains are expected.

**Scheme 2:**
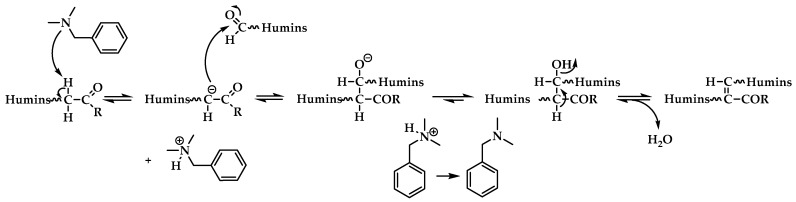
Proposed mechanism of the aldol addition between carbonyl groups of humins and their subsequent dehydration (condensation).

Furthermore, humins present other functionalities that can proceed via other kind of reactions during the thermocuring process. Indeed, the carbonyl functionalities of humins can undergo via aldol addition/condensation with other aldehydes or ketones from humins [34]. Patil et al. proposed the formation of humins via aldol addition and condensation during the acid-catalyzed conversion of 5-hydroxymethylfurfural (HMF) [35]. In fact, the possible intramolecular aldol addition/condensation or between different molecules of humins can be considered an auto-crosslinking of the preformed humins molecules, apart from the reaction that induces humins formation. This reaction can take place between carbonyl groups located in the same molecule of humins (intramolecular reaction) or between carbonyl groups from different molecules (intermolecular reaction). The proposed mechanism is shown in Scheme 2. In humins systems, the relatively acidic hydrogen in the α-position to aldehydes or ketones allow the formation of an enolate in a basic media generated by BDMA. After that, the enolate reacts as a carbon nucleophile and attacks a carbonyl carbon of another aldehyde or ketone function. If possible, this reaction continues via dehydration to give α,β-unsaturated aldehyde or ketone. The overall process is then referred as an aldol condensation. This reaction has also been described at room temperature between small molecules that contains aldehyde groups and ketones using tertiary amines like DBU or diisopropylethylamine (DIPEA) as initiators [36,37]. So, one can anticipate that the aldol addition/condensation could occur in our systems between aldehydes and ketones from the humins simultaneously with the ring-opening copolymerization of the epoxides.

### 3.2. FT-IR Spectroscopy

#### 3.2.1. Raw Materials

The FT-IR spectra of the raw humins, PEGDE, GDE and BDMA are shown in Figure S1. These spectra were used as a reference to elucidate the changes occurred on the functional groups present in humins and in the epoxy compounds during the copolymerization. The assignment of the major bands of humins, in agreement with literature is also summarized in Table S1. The complexity of humins is evidenced by the presence of different functional groups in its structure: Hydroxyl groups, ethers, aldehydes, ketones, carboxylic acids, esters and other carbonyl derivatives linked to furan rings.

The characteristic bands associated to both diepoxy monomers used and to BDMA are assigned in Table S2. The spectrum of GDE contains the characteristic bands associated to the epoxy groups: The C–H stretching vibrations in the oxirane rings appear at 3056 cm^−1^ and 3000 cm^−1^, while the C–O–C stretching vibration is observed at 1253 cm^−1^, the C–O–C asymmetric stretching vibration appears at 906 cm^−1^ and the bending deformation of the epoxy group at 837 cm^−1^ (Figure S1). Furthermore, a band at 3482 cm^−1^ associated to the O–H stretching vibration is attributed to the hydroxyl group. In the case of PEGDE, the same bands associated to the oxirane rings commented for GDE were observed except the O–H stretching vibration.

In the IR spectrum of BDMA, we observe the characteristic C–N stretching vibration related to a tertiary amine present at 1024 cm^−1^. Furthermore, the bands related to the C–H stretching vibrations in the aromatic ring are observed between 3100 and 3000 cm^−1^, whereas the C–H stretching vibrations between 3000 and 2700 cm^−1^ correspond to the C–H stretching vibration of the aliphatic methylene and methyl groups of the amine.

#### 3.2.2. Copolymers Based on Humins–Epoxides Thermosets

**Figure 1:**
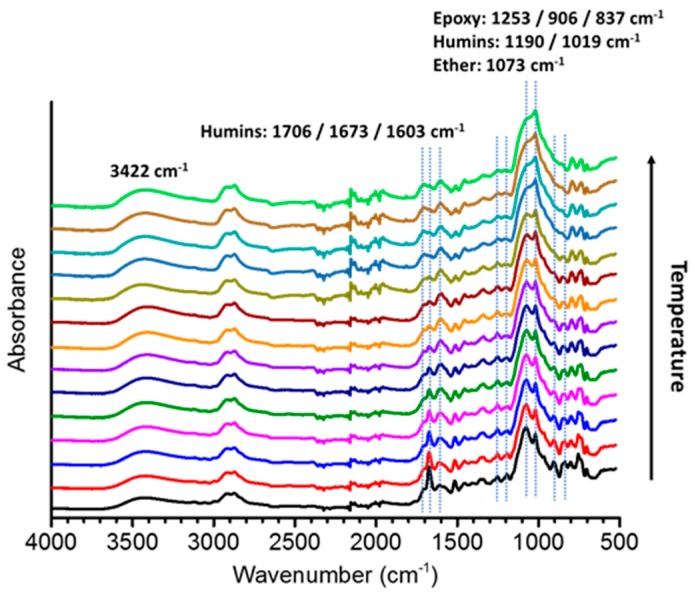
FT-IR spectra evolution during the polymerization of HG40B5 formulation.

The evolution of IR bands during the polymerization of the system HG40B5 is shown in Figure 1 and Figure S2. When the humins were copolymerized with the GDE using BDMA as initiator, the intensity of the bands associated to the epoxide groups decreases progressively until their total disappearance at a temperature of reaction ~130 °C. This observation supports our assumption that the ring-opening of the epoxy groups is the predominant reaction in the humins–epoxy reaction mixtures. Nevertheless, other changes in the spectra are also observed. On the one hand, only one band between 3600 and 3300 cm^−1^ is observed during the curing. This band, that appears at the same wavenumber for the three systems, could be related to the O–H stretching vibration of the hydrogen bonds. Compared with the other two systems, the intensity of this band increases due to the presence of more reactive alkoxide anions generated from GDE during the propagation of the ring-opening polymerization.

In the region between 1800 and 1400 cm^−1^, four main bands are observed: One at 1673 cm^−1^, related to the C=O stretching vibration of the aldehyde groups from humins structure [25,38]. During the curing process, the intensity of this band gradually decreases until their complete disappearance. This observation reinforces the proposed hypothesis that the aldehyde groups of humins would react via aldol additions. In fact, some studies demonstrate that the humins itself are obtained via the aldol addition/condensation reactions between 5-Hydroxymethylfurfural, 5-Methyoxymetylfurfural, levulinic acid and other small organic compounds [17,39]. Another band that supports this hypothesis appears at 1603 cm^-1^. This broad signal is related to the C=C stretching vibration of double bonds conjugated with carbonyl groups. As commented before, the obtained product of an aldol condensation is an α, β-conjugated system. Finally, a band at 1518 cm^−1^ is assigned to the C=C stretching vibration in the furan rings of humins linked to aldehyde groups. The decrease of the intensity of this band during the polymerization reaction means that the chemical environment of these furan moieties of humins is modified. On the other hand, a band at 1508 cm^−1^ associated to the same C=C stretching vibration in other furan rings not linked to aldehydes keeps constant during the polymerization. In fact, this band is clearly observable in the final FT-IR spectra of each system.

**Figure 2:**
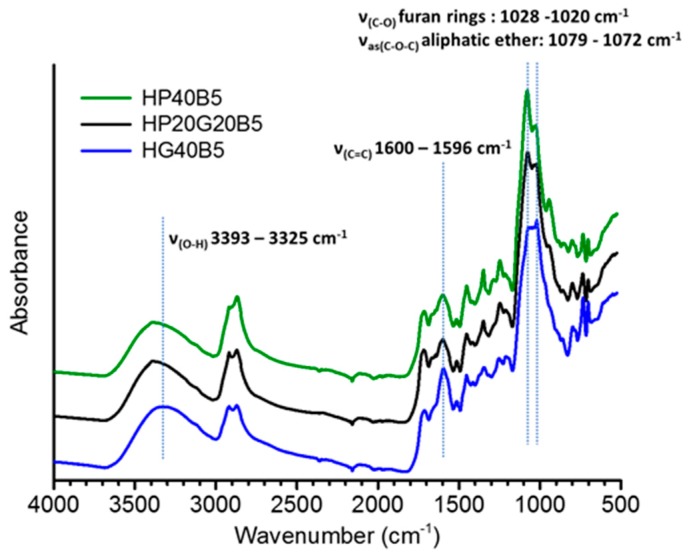
FT-IR spectra of the three thermoset copolymers at the end of the reaction.

Finally, in the region between 1300 and 700 cm^−1^, different bands associated to the humins, the epoxy monomers and the resulting copolymers are detected. Three bands at 768, 804 and 1190 cm^−1^ are assigned to humins (Table S1). While the intensity of the bands at 768 and 1190 cm^−1^ gradually decreases, the band at 804 cm^−1^ associated to the C–H out-of-plane deformation in the furan rings moves to 798 cm^−1^. This variation to a small wavenumber is consistent with the furan substitution in the C_5_ position. Two other bands at 1073 and 1019 cm^−1^ were noted, which were assigned to the C–O–C asymmetric stretching vibration of aliphatic ethers and to the C–O stretching vibration of the furan rings, respectively. As observed in Figure S2, at the end of copolymerization the contribution of the band assigned to the C–O stretching in the furan rings is higher than the contribution of the band attributed to the C–O stretching of the aliphatic ethers, in contrast with the proportion of these peaks at the beginning of reaction. The presence of the hydroxyl groups in GDE (AB_2_ monomer) increments the number of reactive positions during the propagation of the ring-opening polymerization in the HG40B5 system. Hence, the formation of more alkoxide anions increases the degree of branching of the final polymer leading to structures with shorter chains between the crosslinks in which the contribution of humins is higher compared with the epoxide polyether chain [40]. Consequently, once the alkoxides cannot continue the ring-opening propagation due to steric impediments and topological restrictions, the aldol addition/condensation could be favoured. The faster decrease of the band attributed to the aldehyde’s functionalities in humins (1673 cm^−1^) in HG40B5 system confirms this hypothesis. Besides, the band associated to the C=C stretching vibration at around 1600 cm^−1^ shows a higher contribution in the formulation HG40B5. On the other hand, the contribution of the band at~ 1079 cm^−1^ is higher than the band associated to the C-O stretching vibration of the furan rings when more PEGDE was added (Figure 2). PEGDE contains only the oxirane functionalities and the concentration of the propagation species is smaller than in the systems containing the GDE. So, the ring-opening polymerization is slower when increasing the amount of PEGDE and at the same time, the aldol additions are less favoured. Moreover, less hyperbranched polymeric structures are obtained, which results in a higher contribution of the polyether chain in the final polymeric networks.

Two well-defined peaks appear at 1714 and 1732 cm^−1^ when PEGDE is a comonomer (Figure S3). As described in literature [41], these two peaks are associated to the carbonyl groups present in the aliphatic regions of humins, the chemical environment of which has been modified during the curing. The formation of these two peaks at 1715 and 1733 cm^−1^ are also clearly observed for the formulation HP20G20B5, in which a mixture of GDE and PEGDE was used (Figure S4). The observation of these two bands supports the ester formation during the curing process from the carboxylic acid groups of the starting humins.

For the formulation HP20G20B5, the evolution of the IR spectra during the curing is very similar with that of the system HP40B5. Surprisingly, all the peaks that appear in the region between 1650 and 1400 cm^−1^ (assigned to the C–C stretching in aromatic rings, furan rings, C–H asymmetric and symmetric bending in methyl and methylene groups) became thin and well defined during the copolymerization. This observation is related to the different contribution of each monomer in the final composition and proves that all of them are chemically grafted. In other words, different chemical environments were detected by FT-IR during the curing of this formulation [42].

When the FT-IR spectra of the three final thermoset copolymers were compared, the O–H stretching band shows a different value for the formulation HG40B5 (3325 cm^−1^) in contrast with the other two formulations that contains PEGDE (3393 cm^−1^). The higher reactivity expected of GDE vs. PEGDE could help to explain this difference. When both comonomers were combined, the observable O–H stretching vibration is the one attributed to PEGDE contribution. This fact means that PEGDE units are mainly in the external part of the resulting polymers.

One possible side reaction that we proposed was the Diels–Alder reaction between different furan rings in humins. Nevertheless, the typical bands associated to the Diels–Alder adducts at around 1720, 1690 and 868 cm^−1^ are not observed during the reaction evolution neither in the final thermosets. The absence of a clear molecule that can act as a dienophile and the prove that the Diels–Alder cycloaddition between furan rings is not energetically favoured was reported in literature [43].

### 3.3. NMR Spectroscopy

The NMR investigations are presented in two parts. In the first part, we tackle the characterization of humins, the epoxy monomers, BDMA and the initial mixtures. The aim of the second part is to illustrate the covalent linkages that are developed between the epoxides and the humins network. For this purpose, comparative studies between each prepared formulation at t = 0 h and at the end of the curing process (t = 6 h) have been performed.

#### 3.3.1. Raw Materials and Initial Mixtures

**Figure 3:**
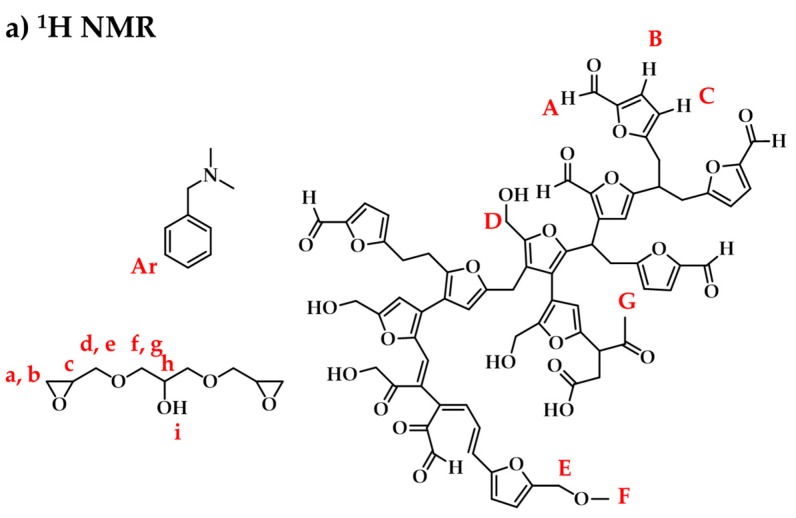

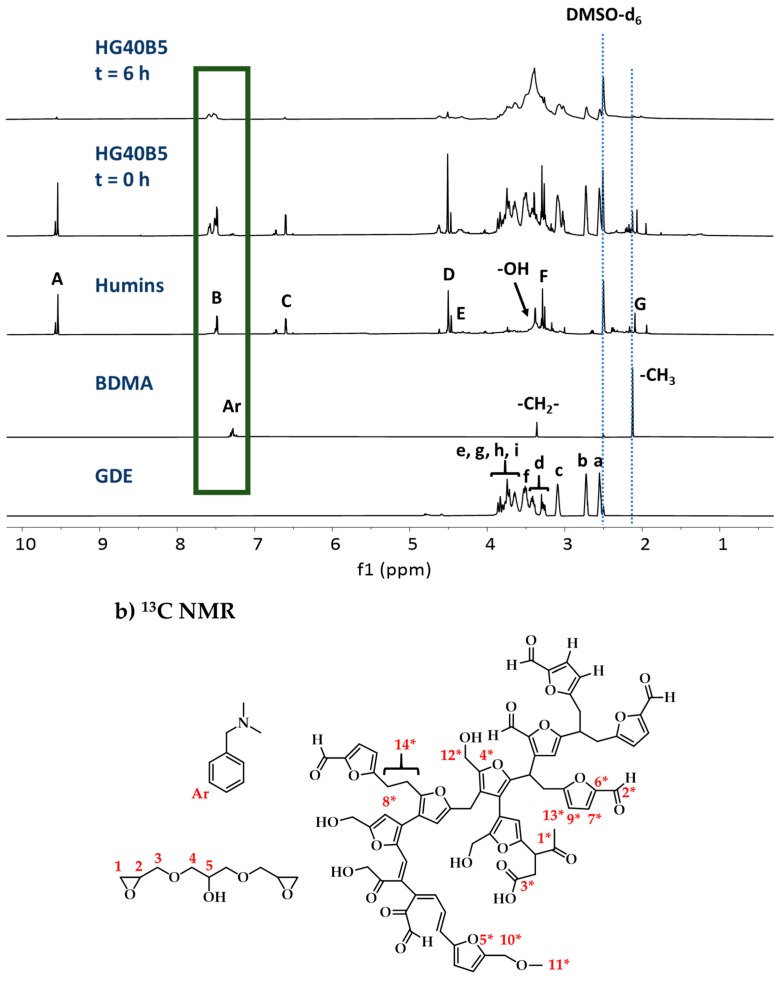

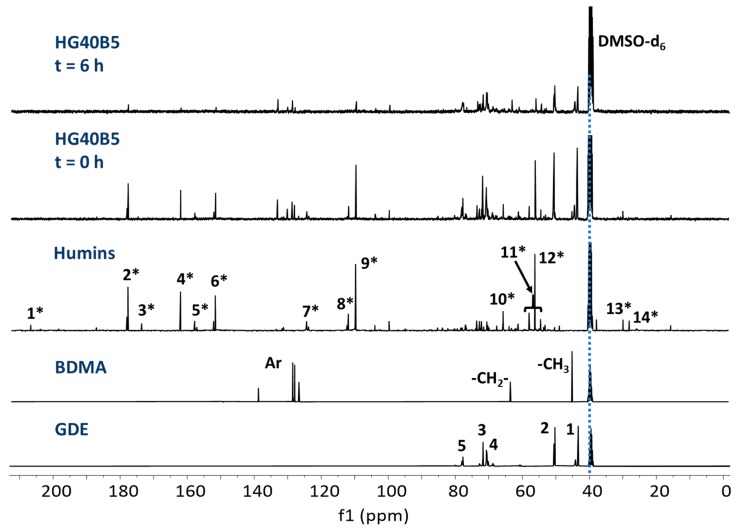
(**a**) ^1^H NMR spectra and (**b**) ^13^C NMR spectra of GDE, BDMA, humins, HG40B5 at t = 0 h and HG40B5 at t = 6 h.

The ^1^H and ^13^C NMR spectra of GDE, BDMA, humins and the formulation HG40B5 at t = 0 h and t = 6 h are shown in Figure 3a,b, respectively. The assignment of the peaks in ^1^H and ^13^C NMR spectra of humins is in agreement with the one described in literature by van Zandvoort et al. [18] Different methylene and methyne units that are linked to the furan rings appear in the region between 2.5 and 4.0 ppm. The signal assigned as F (^1^H δ = 3.30 ppm) is attributed to the end methyl groups of methoxy units linked to the furan rings by a methylene unit (furan-CH_2_-O-CH_3_). A broad peak centred at 3.40 ppm is assigned to the hydroxyl protons. Other broad peaks with smaller intensity can be seen between 4.5 and 5.6 ppm, which demonstrate the complexity of the humins structure. Two singlet signals at 4.45 (H_E_) and 4.50 ppm (H_D_) are associated with the methylene groups that link the furan rings with a methoxy and hydroxyl units. The carbon signals for this methylene groups appear between ^13^C δ = 54 and 58 ppm respectively. In the aromatic region, the protons linked to the C_3_ and C_4_ position of the furan rings appears at 6.60 and 7.50 ppm. The carbon signals attributed to these C_3_ and C_4_ in the furan rings appear in the region between 109 and 125 ppm. The free C_2_ or C_5_ in furan rings, which are expected around 142 ppm are not observed. [18] Furthermore, we can confirm that humins have three aldehyde groups with a different chemical environment, i.e., three different signals between 9.50–9.60 ppm in the ^1^H NMR spectrum correlated with three signals between 178 and 179 ppm in the ^13^C NMR spectrum. In the ^13^C NMR spectrum, a peak at 174 ppm corresponds to the carboxylic acid functionalities present in humins (Signal 3* in Figure 3b and Figure S8).

The assignment of the signals of GDE and BDMA are shown in the same figures. For GDE, the methylene group of the epoxy ring gives rise to two different proton signals centred at ^1^H δ = 2.55 (H_a_) and 2.70 ppm (H_b_), while the methyne group gives a signal centred at 3.10 ppm (H_c_). The methylene protons in the α-position of the oxirane ring give two signals at ^1^H δ = 3.30 (H_d_) and 3.70 ppm (H_e_), respectively. The methyne linked to the hydroxyl group appears at 3.70 ppm (H_h_). The resonances of the carbon atoms from the oxirane rings are found at 43 ppm (C_1_) and 50 ppm (C_2_), whilst the other carbon signals in the ether chain appear between 70 and 78 ppm. In the case of PEGDE, the methylene protons in the α-position of the oxirane ring give two signals at ^1^H δ = 3.25 (H_d_) and 3.70 ppm (H_e_), while this carbon appear at 72 ppm. The polyether chain of PEGDE shows a broad peak between 3.50 and 3.60 ppm in the ^1^H NMR spectrum and two signals in the ^13^C NMR spectrum around 70 ppm (Figures S7 and S8, respectively). Related to BDMA, two singlet signals at ^1^H δ = 2.10 ppm and ^1^H δ = 3.35 ppm are assigned to the methyl and methylene units respectively in its ^1^H NMR spectrum. The protons of the aromatic ring appear between 7.20 and 7.35 ppm. In the ^13^C NMR spectrum, the methyl groups appear at 45 ppm, the methyne group at 63 ppm and the carbons of the aromatic ring are seen between 127 and 139 ppm.

#### 3.3.2. Humins–Epoxy Thermosets

The chemical structures that result from the reaction of humins with diepoxy monomers were investigated by 1D and 2D NMR spectroscopy. Unlike in the FT-IR analyses, the polymerization was carried out in a solution to allow a good homogeneity and chemical interaction of the reagents using DMSO-*d*_6_ as solvent. To perform the NMR, an aliquot of the homogeneous polymerized system in an oven at 130 °C was diluted in DMSO-*d*_6_ then analysed by liquid-state NMR at 25 °C, as described in Section 4.2. As observed in ^1^H NMR spectra acquired at t = 0 h of the three formulations prepared (Figure 3a, Figures S7 and S18), new peaks between 7.50 and 7.60 ppm appeared in the spectra, which are accompanied by a decrease of the aromatic signals of the starting BDMA between 7.20 and 7.35 ppm. Moreover, these new peaks are broader than the ones of BDMA. These quick changes in the chemical shifts of the aromatic protons assigned to BDMA support that this one is covalently attached onto the polymer as a quaternary ammonium salt. Moreover, this fact confirms that the hydroxyl groups from humins act as a proton donor. This observation proves the effectiveness of the selected initiator. Furthermore, the higher intensity of these new peaks in the HG40B5 system also suggests that the hydroxyl groups from GDE can act as a proton donor and start the ring-opening polymerization of the epoxide groups. Moreover, these hydroxyl groups can lead to intra- as well as intermolecular transfer reactions during the propagation, generating new alkoxide anions that further propagates, resulting in structures with a higher degree of cross-linking when GDE was present in the mixtures [44,45].

For the formulation HG40B5, the comparison of the ^1^H and ^13^C NMR spectra of the starting and the final mixture is shown in Figure 3. A clear broadening of the peaks at the end of the curing in both ^1^H NMR and ^13^C NMR spectra was observed, correlated with the increase of the molecular weight during the polymerization. The presence of the peaks related to the oxirane rings at the end of the reaction means that in presence of DMSO and at the 130 °C polymerization temperature, not all the epoxy groups were consumed. A zoom of the ^1^H NMR in the regions of the methylene units linked to furan rings and of the alkoxide units that start the ring opening polymerization is shown in Figure S6; this highlights the new signal at 4.40 ppm that appears in the ^1^H NMR as a result of the crosslinking between humins and bis-epoxide monomers. Furthermore, the decrease of the intensity of the signal at 56.1 ppm related to these methylene units between the furan rings and the hydroxyl groups in humins is also noted in the ^13^C NMR spectra (Figure S6), which corroborates the hypothesis of the initiation step of the ring-opening polymerization by the humins.

**Figure 4:**
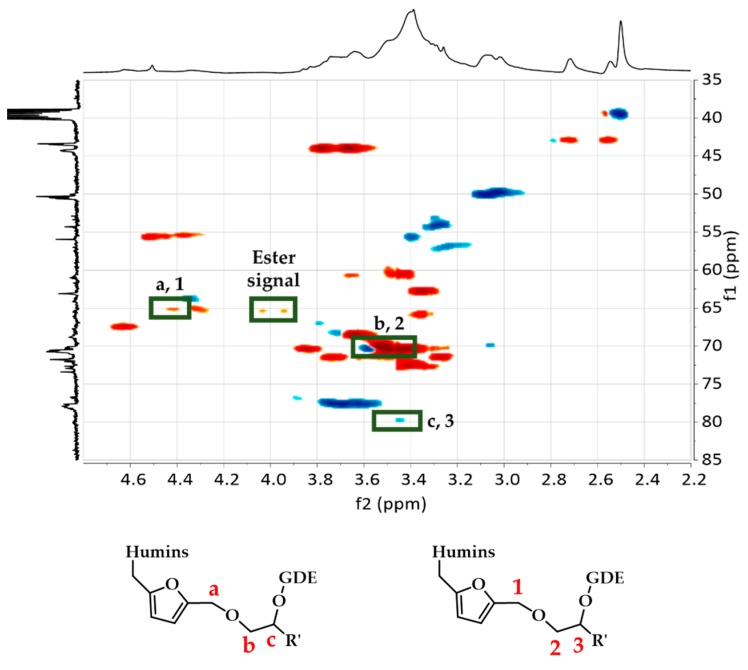
HSQC NMR spectra of HG40B5 at t = 6 h, which shows the cross-peaks attributed to the humins–GDE covalent linkages (green boxes). In the spectra, the blue signals correspond to –CH– and –CH_3_ signals, while the red ones correspond to –CH_2_– signals.

The broad peaks observed in the ^1^H NMR spectra of HG40B5 at the end of the reaction and the low intensity of the new peaks that appeared in the ^13^C NMR make difficult the assignment of the new signals related to the crosslinking between humins and diepoxy monomers by 1 D NMR. Thus, different multidimensional NMR experiments were used to confirm the expected crosslinks. First of all, ^1^J_CH_ correlations were investigated by Heteronuclear Single Quantum Correlation (HSQC) NMR spectroscopy. The HSQC spectra of the formulation HG40B5 at t = 0 h and t = 6 h are shown in Figure S9 and Figure 4, respectively. A cross-peak centred at ~ ^1^H δ=4.40 ppm and at ^13^C δ=65.3 ppm that appears during the curing process corresponds to the methylene group linked to the furan rings in humins chemically bonded to an opened epoxy ring of glycerol diglycidyl ether by an ether linkage: furan-CH_2_-O-CH_2_-CH(R’)-O-GDE (Signal a,1 in Figure 4).

**Figure 5:**
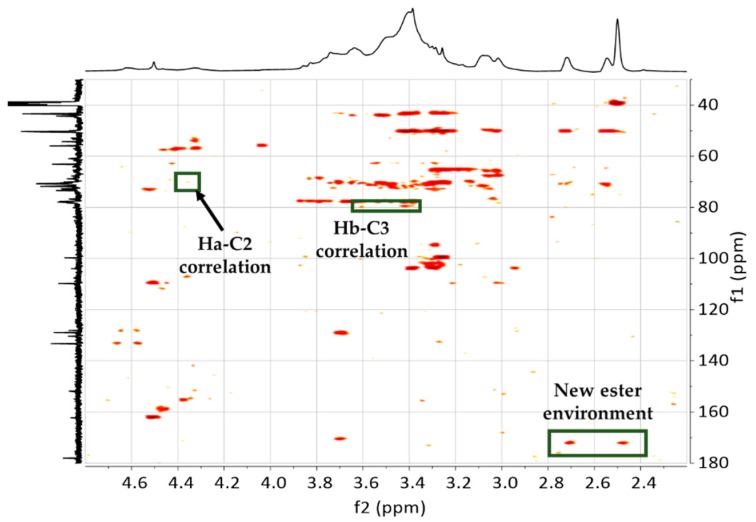
HMBC NMR spectra of HG40B5 at t = 6 h, which shows the additional cross-peaks attributed to the new covalent linkages between humins and epoxides (green boxes).

Furthermore, a methylene signal at 70.3 ppm is correlated to two signals at 3.40 and 3.60 ppm in the HSQC spectra (Signals b,2; Table 2). These protons and carbon signals are related to the methylene group of the opened oxirane ring linked to the humins (signal b,2 in Figure 4). The correlations observed in the Heteronuclear Multiple Bond Correlation (HMBC) spectra at the end of the polymerization: ^1^H δ = 3.40 ppm and ^1^H δ = 3.60 ppm with ^13^C δ = 79.8 ppm (Correlation H_b_-C_3_ in Figure 5) aided us to confirm that this carbon signal could be attributed to the methyne group of the opened oxirane ring. This chemical shift is in agreement with the assignment presented by Vandenberg et al., in which they study the obtention of polyether from glycidol. [44] The cross-peak observed at ^1^H δ = 3.45 ppm and ^13^C δ = 79.8 ppm in the HSQC spectra (signal c, 3 in Figure 4) confirms the presence of this methyne signal in the final structure.

As previously noticed, the carboxylic acid groups found in humins structure could also react with the epoxy groups forming ester linkages. The two new cross-peaks at ^1^H δ = 3.90 ppm and ^13^C δ = 65.5 ppm and ^1^H δ = 4.00 ppm and ^13^C δ = 65.5 ppm that appeared during the polymerization of HG40B5 in the HSQC spectra confirms a new ester environment formed after the ring-opening polymerization of the epoxy rings by the carboxylic acid groups. Nevertheless, the low intensity of these correlations indicates that humins contain a small amount of carboxylic acid groups.

Additionally, the signal that appears at 4.40 ppm during the polymerization attributed to the H_a_ protons (Figure 4) shows new cross-peaks in the HMBC spectrum at t = 6 h (Figure 5), which confirms the covalent connections between the humins and the GDE by an ether linkage. The HMBC spectrum of the formulation HG40B5 at initial time of the polymerization is shown in Figure S10. In the new ether linkage between humins and diepoxy monomers, a ^2^J connection between the H_a_ protons (Figure 5) δ = 4.40 ppm and ^13^C δ = 155.5 ppm (C_5_ in furan rings) appears at the end of the curing. Another cross-peak attributed to a ^3^J connection at ^1^H δ = 4.40 ppm and ^13^C δ = 107.5 ppm (C_4_ in the furan rings) confirms the chemical modification of the hydroxyl groups of humins. At the same time, the observation of a weak ^3^J connection at ^1^H δ = 4.40 ppm and ^13^C δ = 70.3 ppm could be related to the ring-opening of the epoxy by the primary hydroxyl groups of the humins. This ^13^C carbon signal is related to the methylene group of the opened oxirane ring (Signal b,2 in Figure 4), while the ^1^H signal corresponds to the methylene group linked to the furan rings in humins (Signal a,1 in Figure 4). In the final ^13^C NMR spectrum, this peak appears to overlap with the signal assigned as 4 in the ^13^C NMR spectra of GDE (Figure 3b). Nevertheless, the appearance of a small broad peak in the ^13^C NMR spectrum of the cured HP40B5 confirms the chemical shift of the expected methylene group of the opened epoxide linked to the humins (Figure S11).

Besides, the displacement of two cross-peaks from ^1^H δ = 2.35 ppm and ^13^C δ = 174.0 ppm (^2^J) and ^1^H δ = 2.65 ppm and ^13^C δ = 174.0 ppm (^3^J) to ^1^H δ = 2.50 ppm and ^13^C δ = 172.4 ppm (^2^J) and ^1^H δ = 2.70 ppm and ^13^C δ = 172.4 ppm (^3^J) are related to the new esters formed by the ring-opening polymerization of the carboxylic acid groups of humins with the diepoxy monomers.

Regarding the occurrence of GDE homopolymerization, ^1^H NMR spectra of GDE monomer and of GDE homopolymer are shown in Figure S12. Both spectra are practically identical. The main difference between them is that the signals of GDE homopolymer are broader than in the GDE monomer related to the increase of the molecular weight during the formation of GDE homopolymer. Furthermore, the correlations observed in the HSQC spectrum of GDE homopolymer after the curing (t = 6 h, Figure S14) are the same with ones observed in the spectrum at t = 0 h (Figure S13). So, the main cross-peaks at ^1^H δ = 3.50 ppm and ^13^C δ = 70.5 ppm and at ^1^H δ = 3.85 ppm and ^13^C δ = 70.5 ppm suggested that the protons of the methylene signal attributed to the C_2_ in the cross-linked structure between humins and GDE appear overlapped with the methylene signals of the polyether chain. Again, the observation of the cross-peaks at ^1^H δ = 2.55 and 2.70 ppm and ^13^C δ = 43 ppm and ^1^H δ = 3.10 ppm and ^13^C δ = 50 ppm at the end of the polymerization confirms that not all the epoxy groups were consumed in the conditions of samples preparation for the NMR experiments. This evidence helped us to identify the possible structure of our systems. Hence, the formation of hyperbranched polymers in which humins act as a core from which polyether chains grow is the most probable structure [46,47,48].

The homopolymerization of humins with BDMA using the same conditions was also studied. In this case, only the two peaks from the methylene and methyl groups of BDMA changes their chemical displacement (from 2.25 to 2.35 ppm in the case of the methyl groups and from 3.58 to 3.72 ppm in the case of the methylene group, as shown in Figure S15), which corroborates the protonation of the tertiary amine. Apart from this evidence, no other changes were detected by NMR for this system. Moreover, no evidences of the aldol/addition condensation reaction of humins were found by NMR spectroscopy when their evolution was studied in a solution of DMSO-*d*_6_. The use of DMSO-*d*_6_ can affect this reaction because a gradually decrease of the aldehyde band (1673 cm^−1^) combined with the increase of the broad signal at 1603 cm−^1^ (related to the C=C bond formed in the aldol addition) were detected by FT-IR when the polymerizations were carried in bulk (Figures S2–S4) [49,50]. To confirm this hypothesis, the final products obtained in solution were also analysed by FT-IR. As observed in Figure S5, the intensity of the band attributed to the C=C bond around 1600 cm^−1^, which is generated during the aldol condensation, is clearly lower compared with the thermoset obtained in bulk for the formulation HG40B5. Furthermore, a C=O band related to the remainder aldehyde groups of the humins is still observed in the mixture prepared in solution at the end of the polymerization.

**Figure 6:**
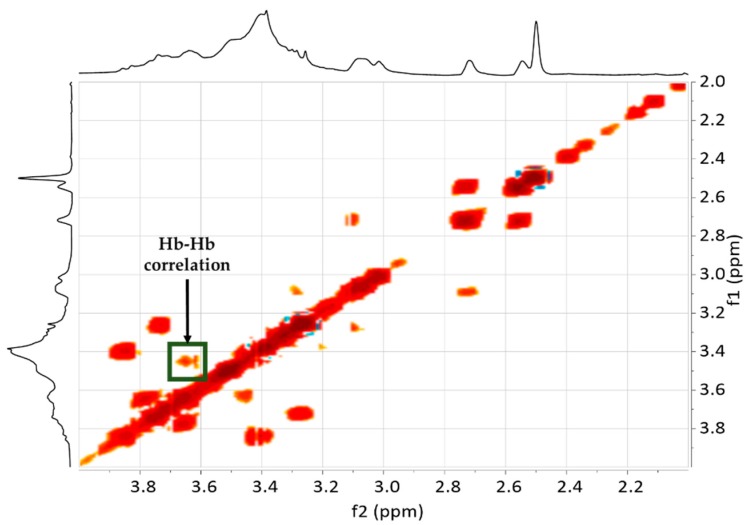
COSY NMR spectra of HG40B5 at t = 6 h, which shows the additional cross-peak attributed to the new covalent linkages between humins and epoxides (green).

On the other hand, a new correlation between two proton signals at 3.40 ppm and 3.60 ppm appeared in the homonuclear correlation spectroscopy (COSY) NMR spectra of the system at t = 6 h (Figure 6). This new correlation, which was not observed at t = 0 h (Figure S16), confirms that these two signals are close in the chemical environment of the molecule. In fact, both protons belong to the methylene units of the opened oxirane ring linked to the humins (H_b_ in Figure 4).

All these predictions are in agreement with an article published by Fréchet group, in which they explore the preparation of hyperbranched polyether from epoxy monomers [51]. To sum up, the assignment of the ^1^H NMR and ^13^C NMR peaks involved in the ether linkage between humins and GDE is shown in Table 2.

The presence of very similar chemical shifts in NMR suggest that the other two formulations follow the same mechanism previously proposed. The evolution of the ^1^H NMR during the curing for the system HP20G20B5 is shown in Figure S18. When both diepoxy where mixed with humins, it seems that the alkoxides have preference to open the epoxy rings of GDE monomer first. In this case, PEGDE act as a reactive diluent to GDE. A reactive diluent is a compound that improves the final conversion by enhancing the kinetics of the propagation reaction [52]. In fact, it will be expected that the addition of the linear flexible PEGDE to a GDE formulation reduces the steric and topological restrictions in the network formation improving the conversion of GDE. The appearance of different broad peaks in the region between 66 and 76 ppm in the ^13^C NMR spectrum of the final formulation, confirms that both epoxy monomers were combined in the resulting polymeric chains (Figure S19). In the same way as in the other two systems, a final conversion of the epoxy groups was not achieved with the conditions described in the experimental section.

**Figure 7:**
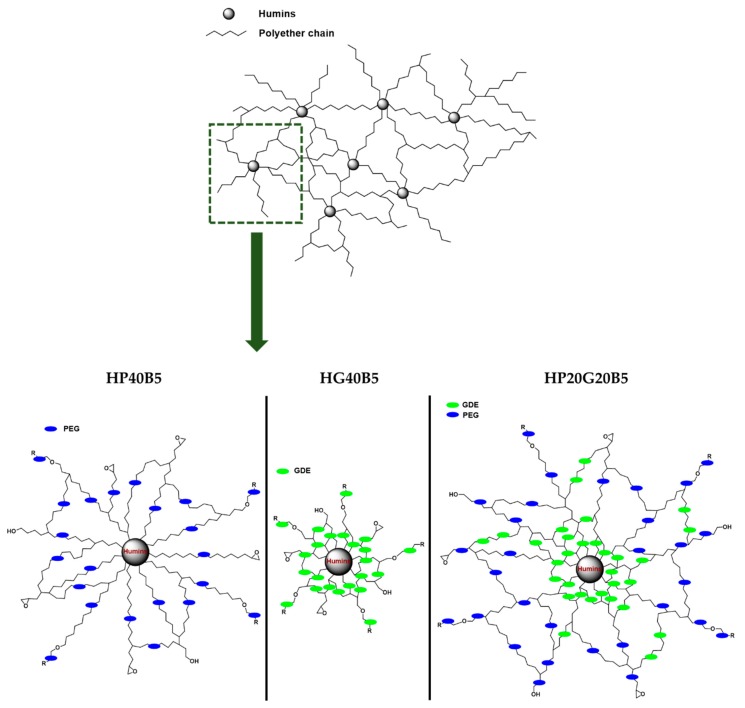
Proposed structures of the obtained polymer networks.

According to the information deduced from FT-IR and NMR studies, the proposed structure of the obtained networks is depicted in Figure 7. In our systems, it will be expected different humins cores are crosslinked by polyether chains, which have grown from the polymerization of bis-epoxide molecules. In the presented structures, humins are the cores of hyperbranched polymers that have hydroxyl and epoxy groups as chain-end. For the formulations HP40G5 and HP20G20B5, the hydroxyl and epoxy end groups are attributed to PEGDE, while in the case of HG40B5, the chain-end groups are attributed to GDE.

## 4. Materials and Methods

### 4.1. Materials

Glycerol diglycidyl ether (GDE, purity: Technical grade), with and epoxy equivalent weight (EEW) of 142 g·equivalent^−1^, and poly(ethylene glycol) diglycidyl ether (PEGDE, average Mn = 500), with an EEW of 275 g·equivalent^−1^, were used as monomers (The epoxy content was calculated by ^1^H NMR). *N*,*N*-Dimethylbenzylamine (BDMA, purity: ≥ 99 %) was used as polymerization initiator. Both monomers and the initiator were purchased from Sigma-Aldrich and were used as received. Humins are directly produced by Avantium Chemicals at their Pilot Plant in Geleen (The Netherlands) by ACD process of carbohydrates into methoxymethylfurfural (MMF). Humins have the appearance of a very viscous, shiny, black bitumen.

### 4.2. Bulk Preparation of Resins

Thermoset bulk preparation was performed following the procedure described below. The components were mixed in the following order: Humins, epoxy monomers and finally BDMA was added. The *w/w* ratio material was chosen for the preparation of the mixtures. The complete homogeneity of the mixture was confirmed before the addition of each component. Once all the components were added, the formulations were heated in an oven at 80 °C for 4 h and then at 130 °C for 1.5 h more to ensure the completion of the cure. Selected times and temperatures for the curing reaction were selected according to the data presented in a previous work recently published, in which we reported on the thermomechanical properties of the humins/PEGDE, humins/GDE and humins/PEGDE/GDE copolymers. ^31^

### 4.3. FT-IR Spectroscopy

The same procedure described for the preparation of bulk resins was applied. Thus, the formulations were heated at 80 °C for 4 h and then at 130 °C for 2 h more. Samples were taken every 30 min and directly analyzed by FT-IR. A NICOLET iS50 FT-IR spectrophotometer with a GladiATR single diamond was used. A spectrum of air was recorded as the background. A total of 32 scans with a resolution of 4 cm^−1^ were accumulated for the background as well as for each sample in the range 4000–500 cm^−1^.

### 4.4. NMR Spectroscopy

1D and 2D NMR spectra (^1^H, ^13^C, COSY, HSQC, HMBC) were recorded on a Bruker Nano Bay AVANCE III HD 400 spectrometer with a direct BBF-H-D-05-Z-probe that operated at 400.17 MHz for ^1^H and 100.32 MHz for ^13^C, respectively. The NMR acquisitions were done at a temperature of 25 °C using DMSO-*d*_6_ as deuterated solvent. The solvent signal of DMSO-*d*_6_ at 2.50 ppm was used as a standard reference.

All NMR experiments were carried out using pulse sequences supplied by the spectrometer manufacturer (BRUKER – TOPSPIN 3.6) and processed via MestreNOVA software (version 14.1.0). ^1^H spectrum was acquired using: 8 KHz spectral width (SW), 64K complex data point, acquisition time (aq) of 4.08s, relaxation delay (D1) of 1s, number of scan (ns) of 32 and a 30° flip angle pulse width. ^13^C{^1^H} spectra were acquired using: 29.7 KHz for SW, 64 K complex data point, 0.9s for aq, D1 of 2s, ns of 4000 and 30° flip angle pulse width. ^1^H decoupling was achieved using WALTZ 16 pulse sequence. Prior to Fourier transformation, the fids were multiplied by an exponential line broadening function of 1 Hz. Gradient Selective COSY (gs-COSY) spectra were obtained with a spectral width of 5.19 KHz in both dimension, 2 K complex data point in F2, 256 t1 increments (8 scans by increment) in F1, 0.19s for aq, D1 of 2s. Prior to Fourier transformation, the data were zero filled in F1. gs-HSQC phase sensitive (echo–antiecho mode) was obtained with a spectral width of 5.19 KHz and 1K complex data point in F2 and a spectral width of 22.1KHz and 256 t1 increment (32 scans by increment) in F1. Others main parameters are: 0.98s for aq, 1.5s for D1. Prior to Fourier transformation, a QSINE window function (SSB =2) was applied in both dimensions and the data were zero filled and linear predicted (NC=32) to 1K data points in F1. gs-HMBC was acquired with a spectral width of 5.19KHz and 1K complex data point in F2 and a spectral width of 29.7KHz and 256 t1 increments (64 scans by increment) in F1. Others main parameters are: 0.39s for aq, 1.5s for D1, 8Hz for J(X-H) long range coupling and 145 Hz for 1j (X-H). Prior to Fourier transformation, a SINE window function (SSB =0) was applied in both dimensions and the data were zero filled and linear predicted (NC=32) to 1K data points in F1.

To do the NMR analyses, the samples were prepared by adding a small quantity of DMSO-*d*_6_ (1g mixture/ 1mL solvent) to the fresh mixture. Then the polymerization was conducted using the same program protocol as in bulk: 4 h at 80 °C followed by 1.5 h at 130 °C. At the end of the polymerization, a viscous mixture was obtained. Thereafter, to perform the NMR tests, an aliquot (or small amount) of the homogeneous polymerized system was diluted in more DMSO-*d*_6_. These diluted samples were analysed by liquid-state NMR at 25 °C.

## 5. Conclusions

The synthesis of completely bio-based thermosets derived from diepoxy monomers and humins were achieved. By means of FT-IR and NMR spectroscopy, the covalent connections between the hydroxyl and the carboxylic acid groups of humins and the oxirane rings of diepoxy monomers were found. We determined that the hydroxyl and the carboxylic acid groups of humins play a key role in the initiation step of the ring-opening polymerization of the diepoxy monomers. It was proved that the presence of BDMA promotes the generation of the alkoxide and carboxylate anions from these hydroxyl and carboxylic acid groups, that subsequently attack the epoxy ring by a nucleophilic addition. Furthermore, we demonstrate that the hydroxyl groups of GDE can act as a proton donor during the propagation of the ring-opening polymerization. On the other hand, the aldol addition/condensation taking place between the carbonyl functionalities of humins once the epoxy ring-opening polymerization has commenced cannot continue due to steric impediments. The obtention of hyperbranched polymeric networks consisting on a core of humins surrounded by an aliphatic polyether in the periphery is envisioned.

## Figures and Tables

**Table 1:** Composition of the formulations and conditions of the two-step curing process.

Formulation	Initial *w*/*w* Ratios: Humins/Diepoxy Monomer/Initiator
HG40B5	Humins (55)/GDE (40)/BDMA (5)
HP40B5	Humins (55)/PEGDE (40)/BDMA (5)
HP20G20B5	Humins (55)/PEGDE (20)/GDE (20)/BDMA (5)

**Table 2:** Assignment of the protons and carbons involved in the resulting covalent ether linkage between humins and GDE in the formulation HG40B5.

Signals	^1^H NMR Chemical Shift (ppm)	^13^C NMR Chemical Shift (ppm)
a, 1	4.40	65.3
b, 2	3.40–3.60	70.3
c, 3	3.45	79.8

## Supplementary Materials

The following are available online: FT-IR and NMR spectra of the raw materials used, the formulations HG40B5, HP40B5, HG20G20B5, GDE and humins homopolymerziation are in the supplementary data for this paper.

## References

[1] Gandini A., Lacerda T.M. (2015). From monomers to polymers from renewable resources: Recent advances. Prog. Polym. Sci..

[2] Sousa A.F., Vilela C., Fonseca A.C., Matos M., Freire C.S.R., Gruter G.J.M., Coelho J.F.J., Silvestre A.J.D. (2015). Biobased Polyesters and Other Polymers from 2,5-Furandicarboxylic Acid: A Tribute to Furan Excellency. Polym. Chem..

[3] Llevot A., Grau E., Carlotti S., Grelier S., Cramail H. (2016). From Lignin-Derived Aromatic Compounds to Novel Biobased Polymers. Macromol. Rapid Commun..

[4] Imre B., García L., Puglia D., Vilaplana F. (2019). Reactive Compatibilization of Plant Polysaccharides and Biobased Polymers: Review on Current Strategies, Expectations and Reality. Carbohydr. Polym..

[5] Zhu Y., Romain C., Williams C.K. (2016). Sustainable Polymers from Renewable Resources. Nature.

[6] Gandini A., Lacerda T.M., Carvalho A.J.F., Trovatti E. (2016). Progress of Polymers from Renewable Resources: Furans, Vegetable Oils, and Polysaccharides. Chem. Rev..

[7] Mashouf Roudsari G., Mohanty A.K., Misra M. (2017). Green Approaches to Engineer Tough Biobased Epoxies: A Review. ACS Sustain. Chem. Eng..

[8] Kumar S., Krishnan S., Mohanty S., Nayak S.K. (2018). Synthesis and Characterization of Petroleum and Biobased Epoxy Resins: A Review. Polym. Int..

[9] Xu J., Li Z., Wang B., Liu F., Liu Y., Liu F. (2019). Recyclable Biobased Materials Based on Diels-Alder Cycloaddition. J. Appl. Polym. Sci..

[10] Haas M.J., McAloon A.J., Yee W.C., Foglia T.A. (2006). A Process Model to Estimate Biodiesel Production Costs. Bioresour. Technol..

[11] Schweizer A. (1938). Caramel and Humin. A Contribution to the Knowledge of the Decomposition Products of Sugars. Recl. Des Trav. Chim. Des Pays Bas.

[12] Schweizer A. (1940). The Composition of the Humins Produced by the Action of Sulphuric Acid on Some Organic Substances. Recl. Des Trav. Chim. Des Pays Bas.

[13] Cheng Z., Everhart J.L., Tsilomelekis G., Nikolakis V., Saha B., Vlachos D.G. (2018). Structural Analysis of Humins Formed in the Brönsted Acid Catalyzed Dehydration of Fructose. Green Chem..

[14] Van Zandvoort I., Koers E.J., Weingarth M., Bruijnincx P.C.A., Baldus M., Weckhuysen B.M. (2015). Structural Characterization of 13C-Enriched Humins and Alkali-Treated 13C Humins by 2D Solid-State NMR. Green Chem..

[15] Herzfeld J., Rand D., Matsuki Y., Daviso E., Mak-Jurkauskas M., Mamajanov I. (2011). Molecular Structure of Humin and Melanoidin via Solid State NMR. J. Phys. Chem. B.

[16] Sumerskii I.V., Krutov S.M., Zarubin M.Y. (2010). Humin-like Substances Formed under the Conditions of Industrial Hydrolysis of Wood. Russ. J. Appl. Chem..

[17] Patil S.K.R., Heltzel J., Lund C.R.F. (2012). Comparison of Structural Features of Humins Formed Catalytically from Glucose, Fructose, and 5-Hydroxylmethylfurfuraldehyde. Energy Fuels.

[18] Van Zandvoort I., Wang Y., Rasrendra C.B., Van Eck E.R.H., Bruijnincx P.C.A., Heeres H.J., Weckhuysen B.M. (2013). Formation, Molecular Structure, and Morphology of Humins in Biomass Conversion: Influence of Feedstock and Processsing Conditions. ChemSusChem.

[19] Tsilomelekis G., Orella M.J., Lin Z., Cheng Z., Zheng W., Nikolakis V., Vlachos D.G. (2016). Molecular Structure, Morphology and Growth Mechanisms and Rates of 5-Hydroxymethyl Furfural (HMF) Derived Humins. Green Chem..

[20] Shi N., Liu Q., Ju R., He X., Zhang Y., Tang S., Ma L. (2019). Condensation of α-Carbonyl Aldehydes Leads to the Formation of Solid Humins during the Hydrothermal Degradation of Carbohydrates. ACS Omega.

[21] Hoang T.M.C., van Eck E.R.H., Bula W.P., Gardeniers J.G.E., Lefferts L., Seshan K. (2015). Humin Based By-Products from Biomass Processing as a Potential Carbonaceous Source for Synthesis Gas Production. Green Chem..

[22] Filiciotto L., Balu A.M., Romero A.A., Rodríguez-Castellón E., Van der Waal J.C., Luque R. (2017). Bening-by-Design Preparation of Humin-Based Iron Oxide Initiatoric Nanocomposites. Green Chem..

[23] Kang S., Fu J., Deng Z., Jiang S., Zhong G., Xu Y., Guo J., Zhou J. (2018). Valorization of Biomass Hydrolysis Waste: Activated Carbon from Humins as Exceptional Sorbent for Wastewater Treatment. Sustainability.

[24] Chernysheva D.V., Chus Y.A., Klushin V.A., Lastovina T.A., Pudova L.S., Smirnova N.V., Kravchenko O.A., Chernyshev V.M., Ananikov V.P. (2018). Sustainable Utilization of Biomass Refinery Wastes for Accessing Activated Carbons and Supercapacitor Electrode Materials. ChemSusChem.

[25] Tosi P., Van Klink G.P.M., Celzard A., Fierro V., Vincent L., de Jong E., Mija A. (2018). Auto-Crosslinked Rigid Foams Derived from Biorefinery Byproducts. ChemSusChem.

[26] Mija A., de Jong E., van der Waal J.C., van Klink G. (2017). Humins Containing Foam.

[27] Blank W.J., He Z.A., Picci M. (2002). Catalyssis of the Epoxy-Carboxyl Reaction. J. Coat. Technol..

[28] Vidil T., Tournilhac F., Musso S., Robisson A., Leibler L. (2016). Control of Reactions and Network Structures of Epoxy Thermosets. Prog. Polym. Sci..

[29] Ellis B., Ellis B. (1993). Introduction to the Chemistry, Synthesis, Manufacture and Characterization of Epoxy Resins. Chemistry and Technology of Epoxy Resins.

[30] Pin J.-M., Guigo N., Vincent L., Sbirrazzuoli N., Mija A. (2015). Copolymerization as a Strategy to Combine Epoxidized Linseed Oil and Furfuryl Alcohol: The Design of a Fully Bio-based Thermoset. ChemSusChem.

[31] Dinu R., Mija A. (2019). Polyfuranic Frame Networks with Elastomeric Behaviour Based on Humins Biorefinery By-Products. Green Chem..

[32] Rozenberg B.A. (1986). Thermodynamics and Mechanism of Reactions of Epoxy Oligomers with Amines. Adv. Polym. Sci..

[33] Ashcroft W.R., Ellis B. (1993). Curing Agents for Epoxy Resins. Chemistry and Technology of Epoxy Oligomers with Amines.

[34] Braun M., Mahrwald R. (2008). Fundamentals and Transition-State Models. Aldol Additons of Group 1 and 2 Enolates. Modern Aldol Reactions.

[35] Patil S.K.R., Lund C.R.F. (2011). Formation and Growht of Humins via Aldol Addition and Condensation during Acid-Catalyzed Conversion of 5-Hydroxymethylfurfural. Energy Fuels.

[36] List B., Mahrwald R. (2008). Amine-Catalyzed Aldol Reactions. Modern Aldol Reactions.

[37] Markert M., Mulzer M., Schetter B., Mahrwald R. (2007). Amine-Catalyzed Direct Aldol Addition. J. Am. Chem. Soc..

[38] Muralidhara A., Tosi P., Mija A., Sbirrazzuoli N., Len C., Engelen V., De Jong E., Marlair G. (2018). Insights on Thermal and Fire Hazards of Humins in Support of Their Sustainable Use in Advanced Biorefineries. ACS Sustain. Chem. Eng..

[39] Heltzel J., Patil S.K.R., Lund C.R.F., Schlaf M.Z., Zhang Z.C. (2016). Humin Formation Pathways. Reactions and Mechanisms in Thermocatalytic Biomass Conversion II.

[40] Guzmán D., Ramis X., Fernández-Francos X., De la Flor S., Serra A. (2018). Preparation of New Biobased Coatings from a Triglycidyl Eugenol Derivative through Thiol-Epoxy Click Reaction. Prog. Org. Coat..

[41] Pin J.-M., Guigo N., Mija A., Vincent L., Sbirrazzuoli N., Van der Waal J.C., De Jong E. (2014). Valorization of Biorefinery Side-Stream Products: Combination of Humins with Polyfurfuryl Alcohol for Composite Elaboration. ACS Sustain. Chem. Eng..

[42] Munteanu S.B., Vasile C. (2005). Spectral and Thermal Characterization of Styrene-Butadiene Copolymers with Different Architectures. J. Optoelectron. Adv. Mater..

[43] Montero A.L., Montero L.A., Martínez R., Spange S. (2006). Ab Initio Modelling of Crosslinking in Polymers. J. Mol. Struct. THEOCHEM.

[44] Vandenberg E.J. (1985). Polymerization of Glycidol and Its Derivatives: A New Rearrangement Polymerization. J. Polym. Sci. Pol. Chem..

[45] Sunder A., Hanselmann R., Frey H., Mülhaupt R. (1999). Controlled Synthesis of Hyperbranched Polyglycerols by Ring-Opening Multibranching Polymerization. Macromolecules.

[46] Gao C., Yan D. (2004). Hyperbranched Polymers: From Synthesis to Applications. Prog. Polym. Sci..

[47] Voit B.I., Lederer A. (2009). Hyperbranched and Highly Branched Polymer Architectures—Synthetic Strategies and Major Characterization Aspects. Chem. Rev..

[48] Chen S., Xu Z., Zhang D. (2018). Synthesis and Application of Epoxy-Ended Hyperbranched Polymers. Chem. Eng. J..

[49] Huang Y. (2000). Concerning the Solvent Effect in the Aldol Condensation. Monatsh. Chem..

[50] Kapoor M., Majumber A.B., Nath Gupta M. (2015). Promiscuous Lipase-Catalyzed C-C Bond Formation Reactions Between 4 Nitrobenzaldehyde and 2-Cyclohexen-1-One in Biphasic Medium: Aldol and Morita-Baylis-Hillman Adduct Formations. Catal. Lett..

[51] Emrick T., Chang H., Fréchet J.M.J. (2000). The Preparation of Hyperbranched Aromatic and Alipathic Polyether Epoxies by Chloride-catalyzed Proton Transfer Polymerization from ABn and A2 + B3 Monomers. J. Polym. Sci. Pol. Chem..

[52] Ahn K.D., Kim M.H. (2012). Enhanced Cationic Photocuring of Epoxides with Styrene Oxide as a Reactive Diluent. Prog. Org. Coat..

