# Supplementary material for: Synthesis of Resins Using Epoxies and Humins as Building Blocks: A Mechanistic Study Based on In-Situ FT-IR and NMR Spectroscopies
Source: Molecules. 2019 Nov 14;24(22):4110. doi: 10.3390/molecules24224110 (PMC6891716; doi:10.3390/molecules24224110)
Supplement: Supplementary file 1 [file molecules-24-04110-s001.pdf]

Supplementary Materials: Synthesis of resins using epoxies and humins as building blocks: A mechanistic study based on in-situ FT-IR and NMR spectroscopies

Xavier Montané [1], Roxana Dinu [1] and Alice Mija [1,*]

CONTENTS OF THE SUPPORTING INFORMATION

1. **Complementary figures for FT-IR spectroscopy investigations**

Figure S1. FT-IR spectra of the used initiator (BDMA) and raw materials: GDE, PGDE and humins.

Table S1. Assignments of major bands on FT-IR spectra of humins.

Table S2. Assignments of major bands on FT-IR spectra of GDE, PGDE and BDMA.

Figure S2. FT-IR spectra evolution during the copolymerization of HG40B5.

Figure S3. FT-IR spectra evolution during the copolymerization of HP40B5.

Figure S4. FT-IR spectra evolution during the copolymerization of HP20G20B5.

Figure S5. FT-IR spectra of the final HG40B5 copolymers obtained in bulk and in solution.

2. **Complementary figures for NMR investigations**

Figure S6. Zoom of a) ^{1}H NMR and b) ^{13}C NMR spectra of HG40B5 at t = 0 h and t = 6 h.

Figure S7. ^{1}H NMR spectra of PGDE, BDMA, humins, HP40B5 at t = 0 h and HP40B5 at t = 6 h.

Figure S8. ^{13}C NMR spectra of PGDE, BDMA, humins, HP40B5 at t = 0 h and HP40B5 at t = 6 h.

Figure S9. a) **HSQC NMR spectra of HG40B5 at t = 0 h. b)** zoom of the region between 3.0 – 5.0 ppm in ^{1}H NMR and 55.0 – 75.0 ppm in ^{13}C NMR **of the same HSQC NMR spectra. In the spectra, the blue signals correspond to -CH- and -CH_3 signals, while the red ones correspond to -CH_2- signals.**

Figure S10. a) HMBC NMR spectra of HG40B5 at t = 0 h. b) Zoom between 2.10 - 2.90 ppm in ^{1}H NMR and between 171.0 – 179.0 ppm in ^{13}C NMR region of the same HMBC NMR spectra.

Figure S11. ^{13}C NMR spectra of HP40B5 at t = 0 h and t = 6 h.

Figure S12. ^{1}H NMR spectra of GDE monomer and GDE homopolymer at t = 6 h.

Figure S13. HSQC NMR spectra of GDE homopolymer at t = 0 h. In the spectra, the blue signals correspond to -CH- and -CH_3 signals, while the red ones correspond to -CH_2- signals.

Figure S14. HSQC NMR spectra of GDE homopolymer at t = 6 h. In the spectra, the blue signals correspond to -CH- and -CH_3 signals, while the red ones correspond to -CH_2- signals.

Figure S15. ^1H NMR spectra of humins with BDMA at t = 0 h and the same mixture at t = 6 h.

Figure S16. COSY NMR spectra of HG40B5 at t = 0 h.

Figure S17. COSY NMR spectra of GDE95B5 (DGE homopolymer) at t = 6 h.

Figure S18. ^1H NMR spectra of HP20G20B5 at t = 0 h and at t = 6 h.

Figure S19. ^{13}C NMR spectra of HP20G20B5 at t = 0 h and t = 6 h.

1. Complementary figures for FT-IR spectroscopy investigations

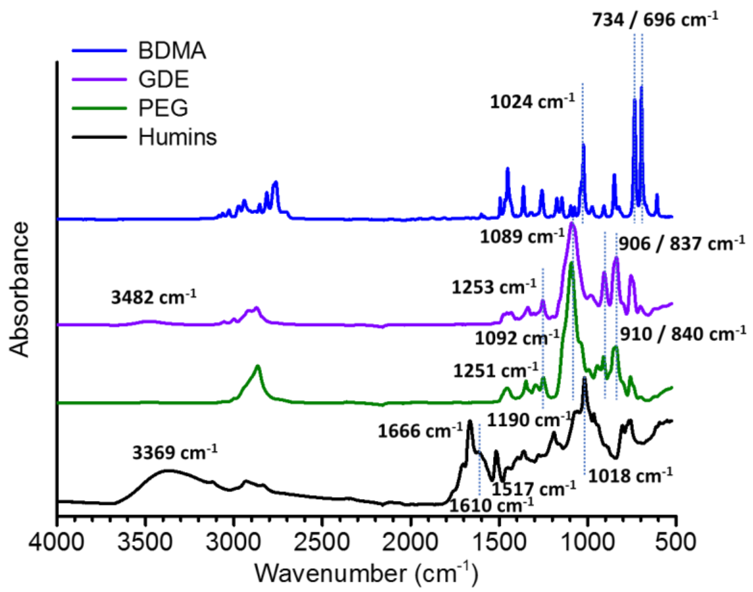

Figure S1. FT-IR spectra of the used initiator (BDMA) and raw materials: GDE, PGDE and humins.

Table S1. Assignment of major bands on FT-IR spectra of humins.

Wavenumber (cm^{-1})	Assignment
3369	O-H stretching vibration of associated -OH by hydrogen bonding
3120	-(C=**C)-H** asymmetric/symmetric stretching vibration
2930	C-H ($-CH_2-$) asymmetric stretching vibration in aliphatic methylene units
2837	C-H ($-CH_2-$) symmetric stretching vibration in aliphatic methylene units
1702	-C=O stretching vibration of acids, esters and conjugated carbonyl groups
1666	-C=O stretching vibration of aldehyde groups
1617	-C=C- stretching vibration conjugated to -C=O
1580-1500	-C=C- stretching vibration in furan rings
1517	-C=C- stretching vibration in furan rings linked to aldehyde groups
1490-1410	C-H asymmetric bending deformation in $-CH_3$; C-H asymmetric and symmetric bending deformation in $-CH_2-$
1360	C-H symmetric bending deformation in $-O-CH_3$
1190	-C-C- asymmetric stretching vibration in furan rings
1018	-C-O- stretching vibration in furan rings
804	-(C=**C)-H** wagging out-of-plane in furan rings (bending)
768	-(C=**C)-H** wagging out-of-plane in furan rings (bending)

Table S2. Assignments of major bands on FT-IR spectra of GDE, PGDE and BDMA.

Wavenumber (cm^{-1})	Assignment	Compound
3482	O-H stretching vibration	GDE
3100-3000	C-H stretching vibration (aromatic)	BDMA
3056	C-H stretching vibration (-CH-); epoxy group	PGDE, GDE
3000	C-H stretching vibration (-CH-); epoxy group	GDE
3000-2700	C-H stretching vibration (aliphatic)	PGDE, GDE, BDMA
2993	C-H stretching vibration (-CH-); epoxy group	PGDE
1490-1410	C-H asymmetric and symmetric bending deformation in $-CH_2-$	PGDE, GDE
1253	C-O-C symmetric stretching vibration of oxirane group; epoxy group	GDE
1251	C-O-C symmetric stretching vibration of oxirane group; epoxy group	PGDE
1092	C-O-C asymmetric stretching vibration; ether linkage	PGDE
1089	C-O-C asymmetric stretching vibration; ether linkage	GDE
1024	C-N stretching vibration; aliphatic tertiary amine	BDMA
910	C-O-C asymmetric stretching vibration of oxirane group; epoxy group	PGDE
906	C-O-C asymmetric stretching vibration of oxirane group; epoxy group	GDE
840	C-O-C bending deformation of oxirane group; epoxy group	PGDE
837	C-O-C bending deformation of oxirane group; epoxy group	GDE
758	C-H wagging out-of-plane in epoxy ring (bending)	PGDE
755	C-H wagging out-of-plane in epoxy ring (bending)	GDE
734	C-H out-of-plane bending deformation in the aromatic ring	BDMA
696	C-H out-of-plane bending deformation in the aromatic ring	BDMA

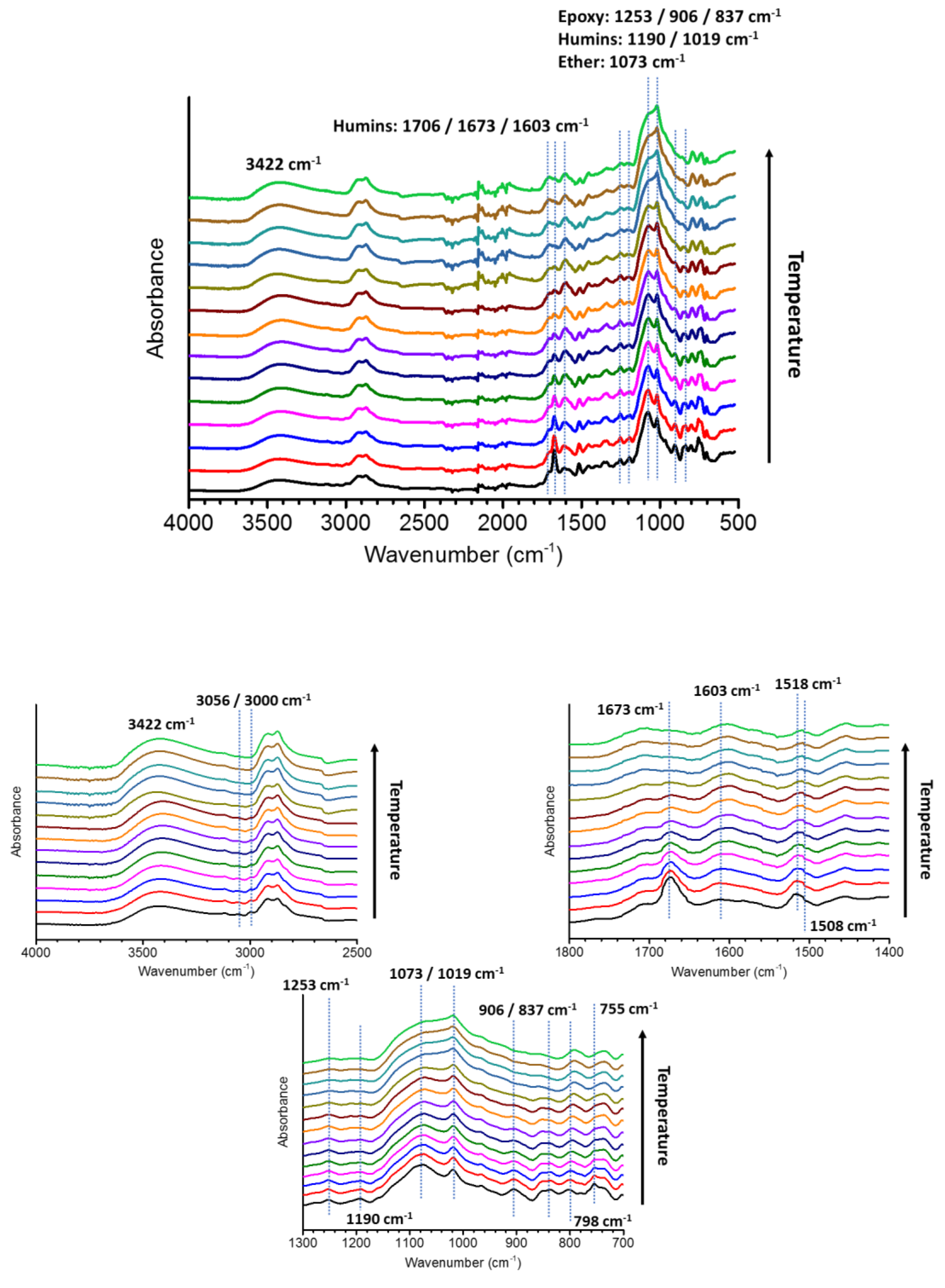

Figure S2. FT-IR spectra evolution during the polymerization of HG40B5.

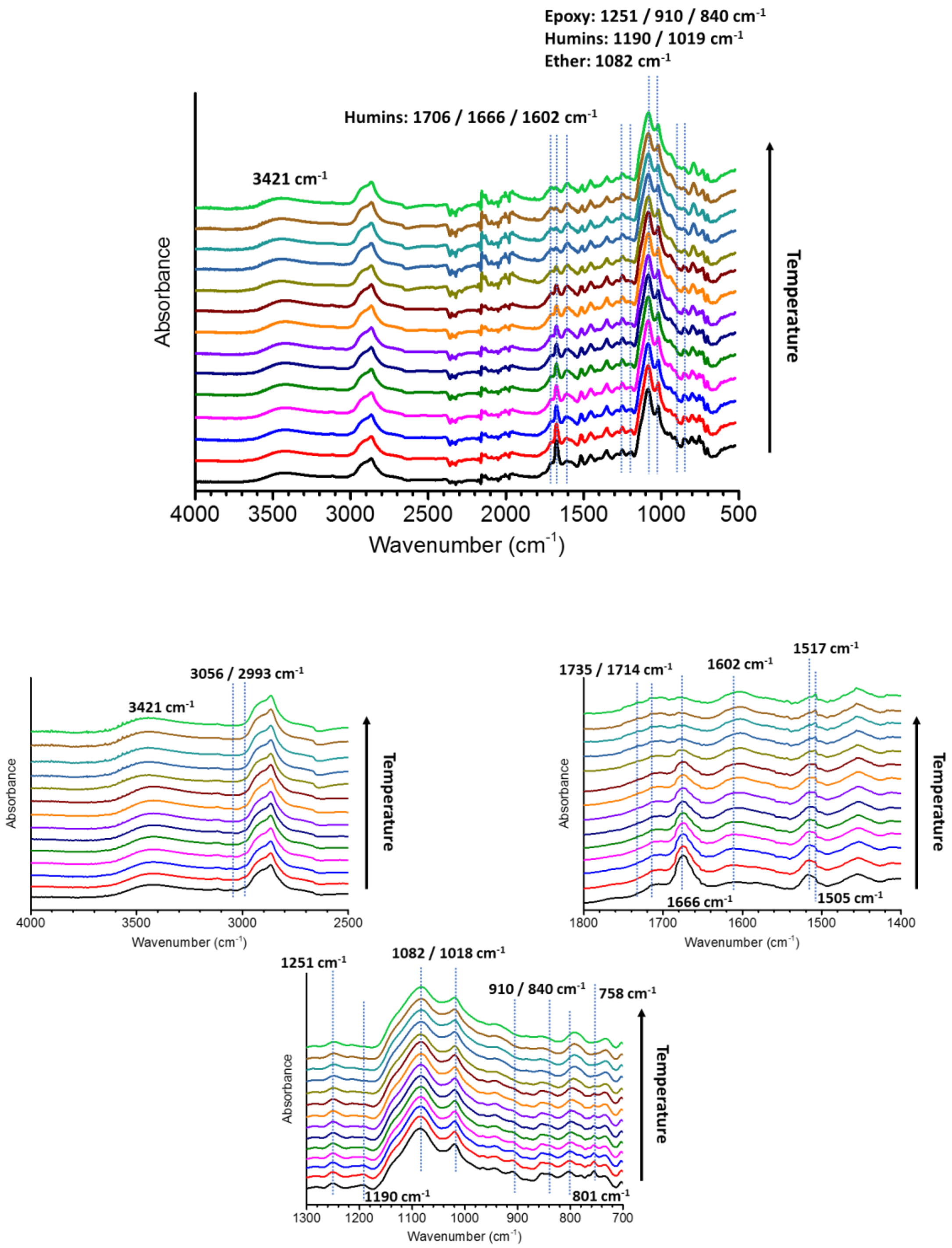

Figure S3. FT-IR spectra evolution during the polymerization of HP40B5.

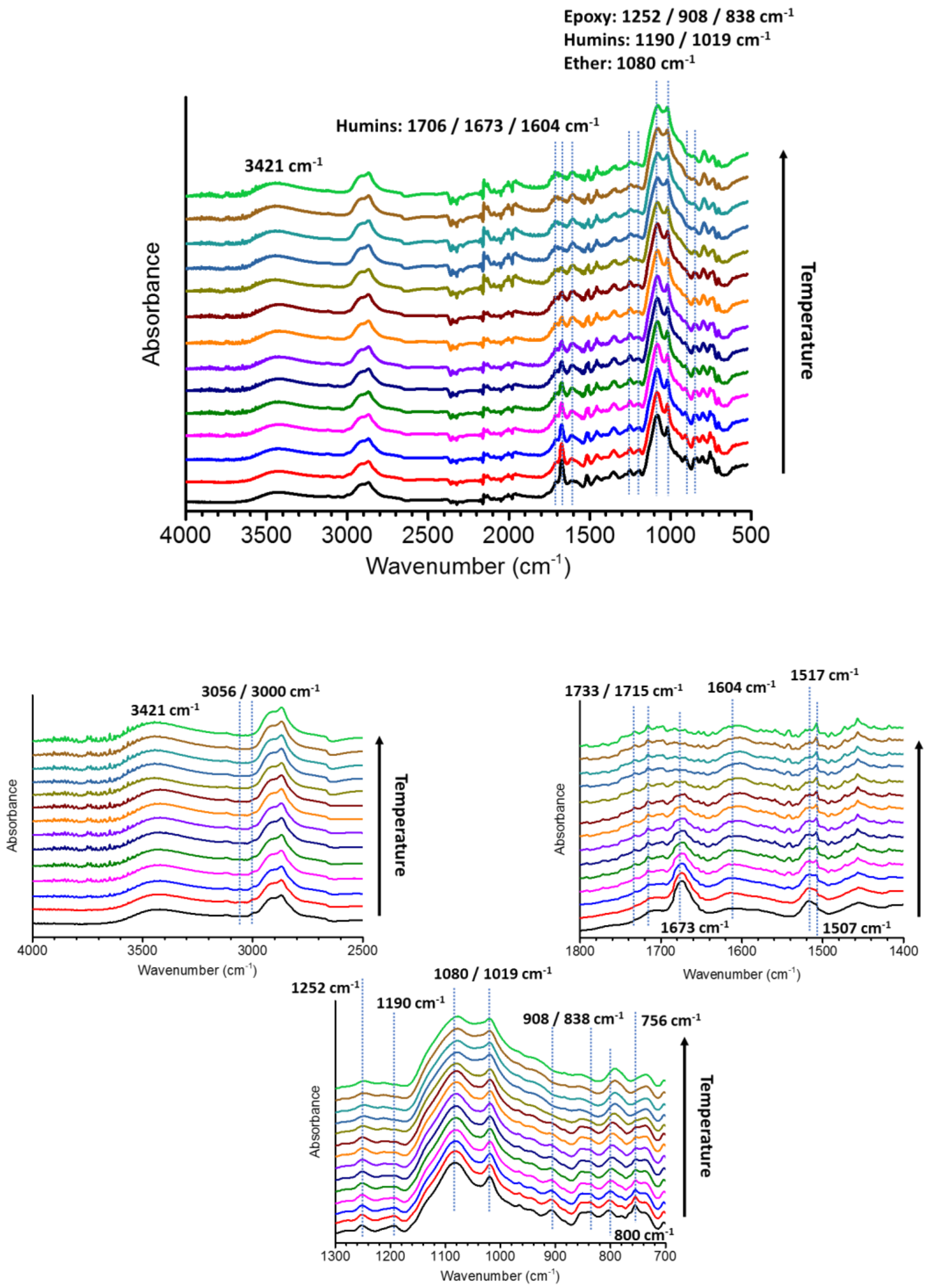

Figure S4. FT-IR spectra evolution during the polymerization of HP20G20B5.

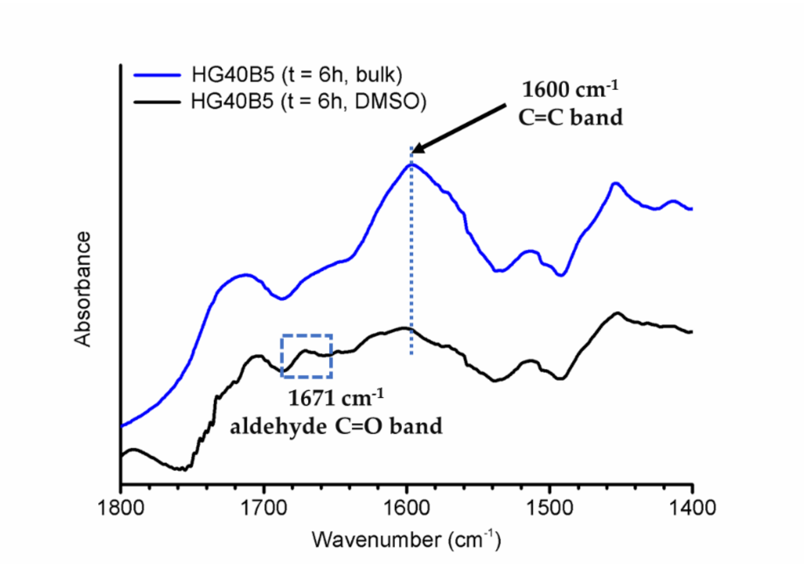

Figure S5. FT-IR spectra of the final HG40B5 copolymers obtained in bulk and in solution.

2. Complementary figures for NMR investigations

a) ^{1}H NMR

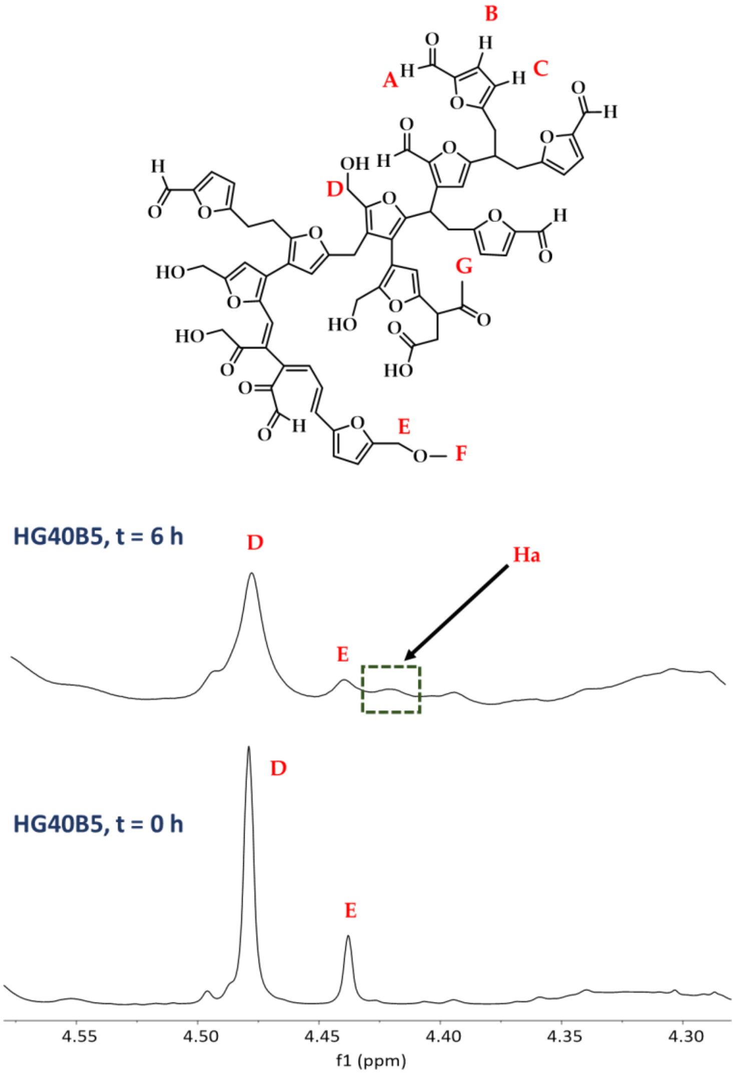

b) ^{13}C NMR

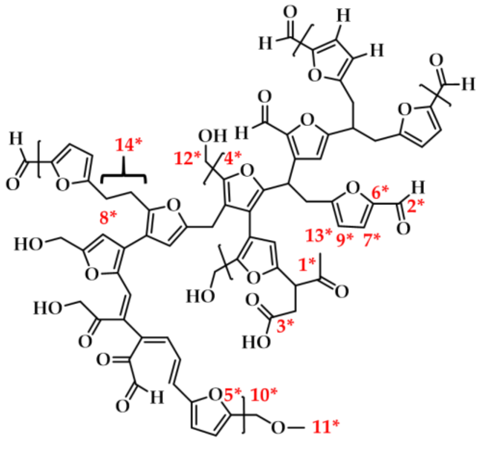

b) ^{13}C NMR

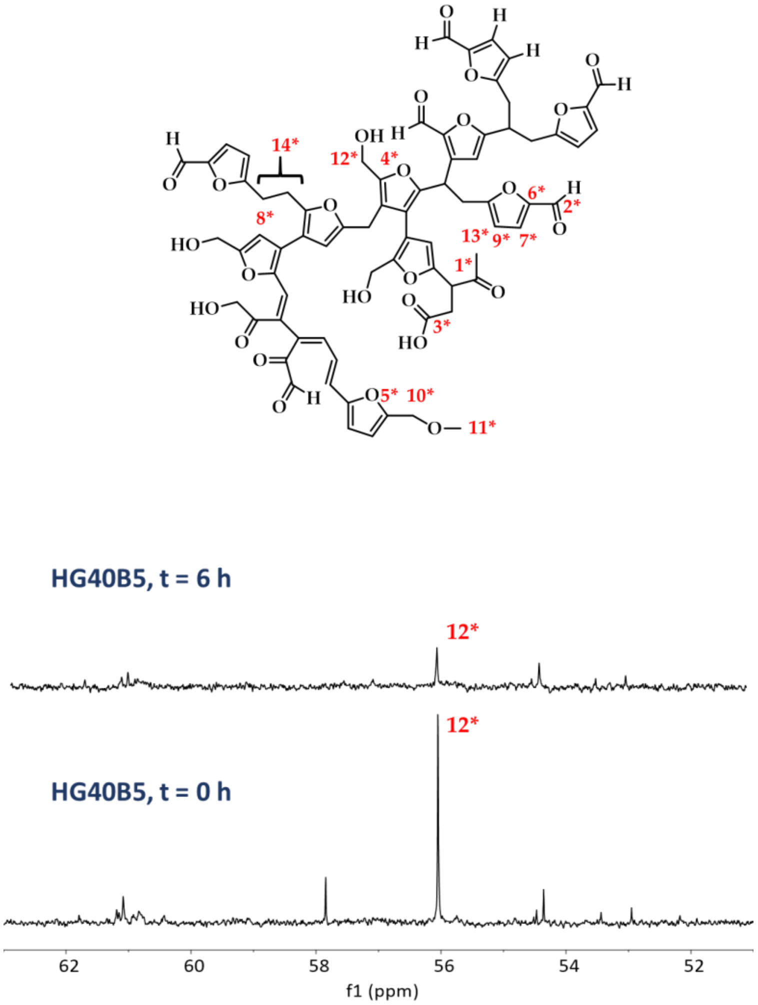

Figure S6. Zoom of a) ^{1}H NMR and b) ^{13}C NMR spectra of HG40B5 at t = 0 h and t = 6 h.

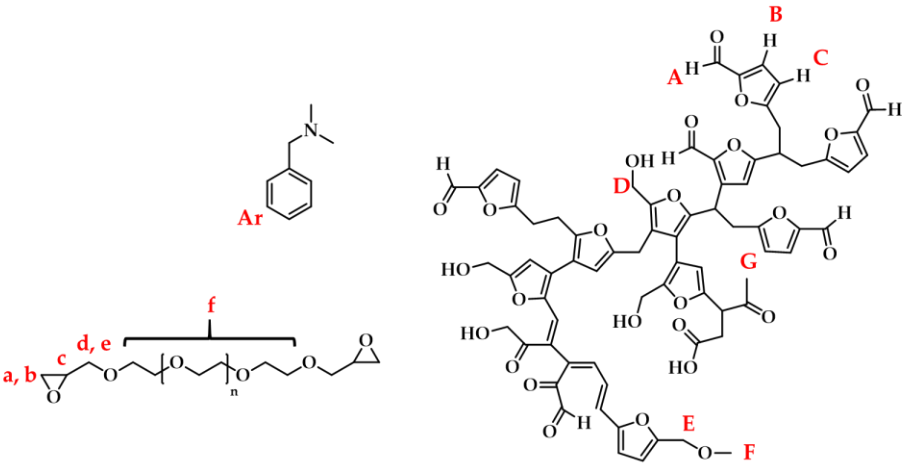

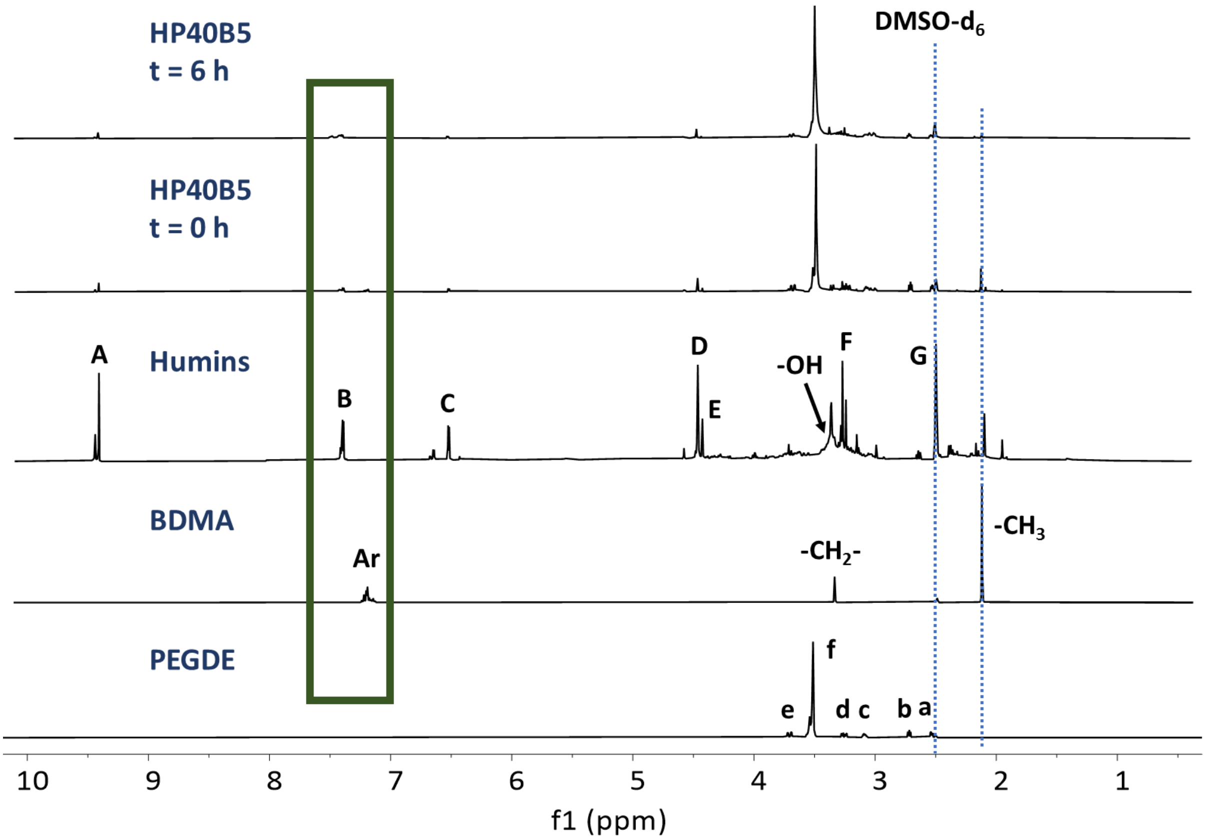

Figure S7. ^{13}C NMR spectra of PGDE, BDMA, humins, HP40B5 at t = 0 h and HP40B5 at t = 6 h.

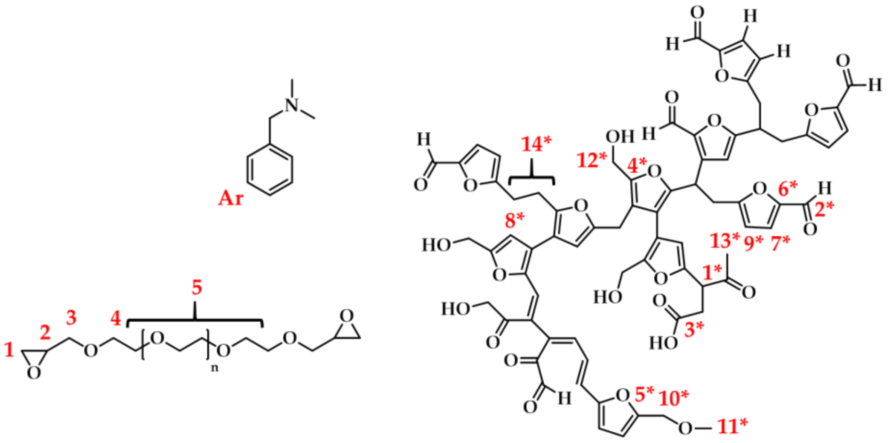

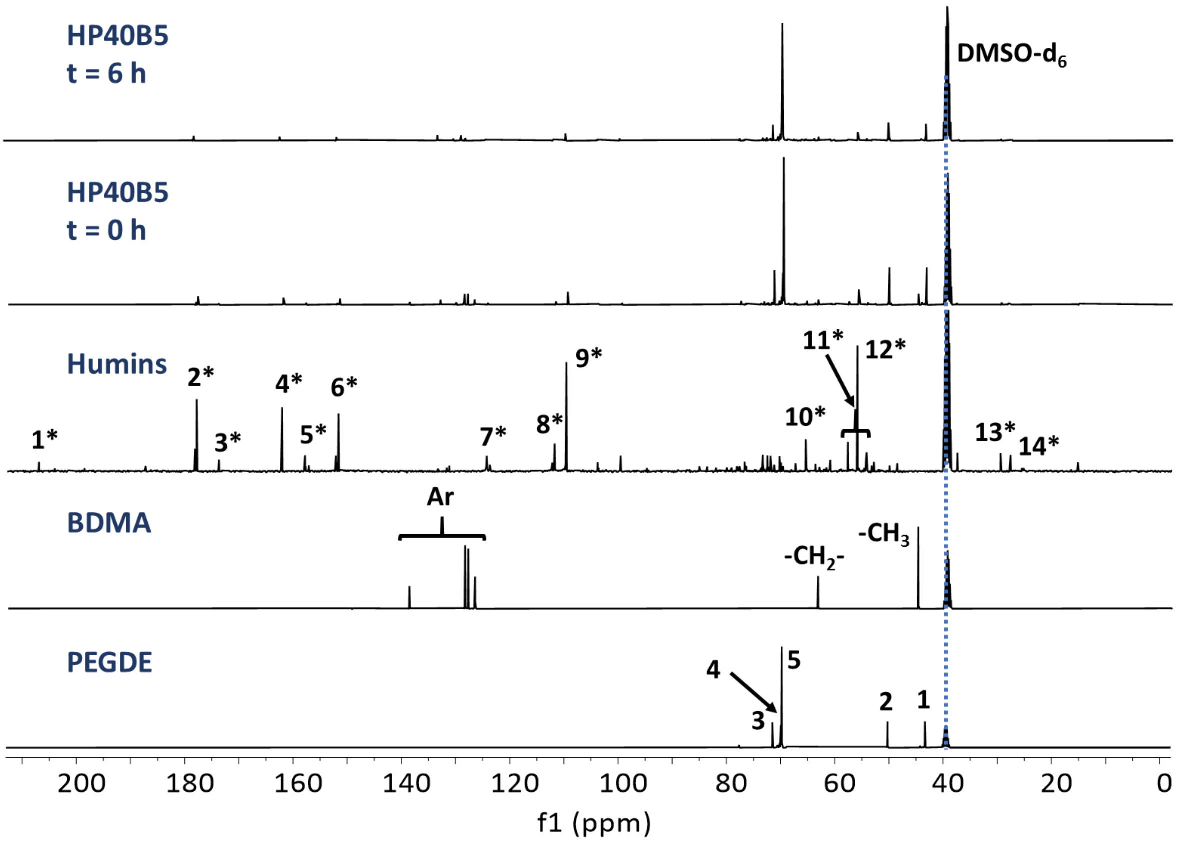

Figure S8. ^{13}C NMR spectra of PGDE, BDMA, humins, HP40B5 at t = 0 h and HP40B5 at t = 6 h.

a) HSQC spectra

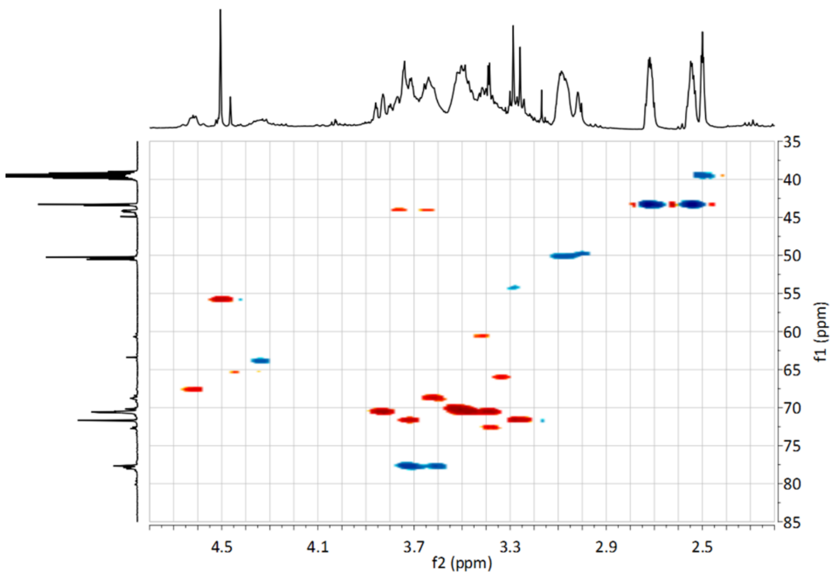

b) Zoom of HSQC spectra

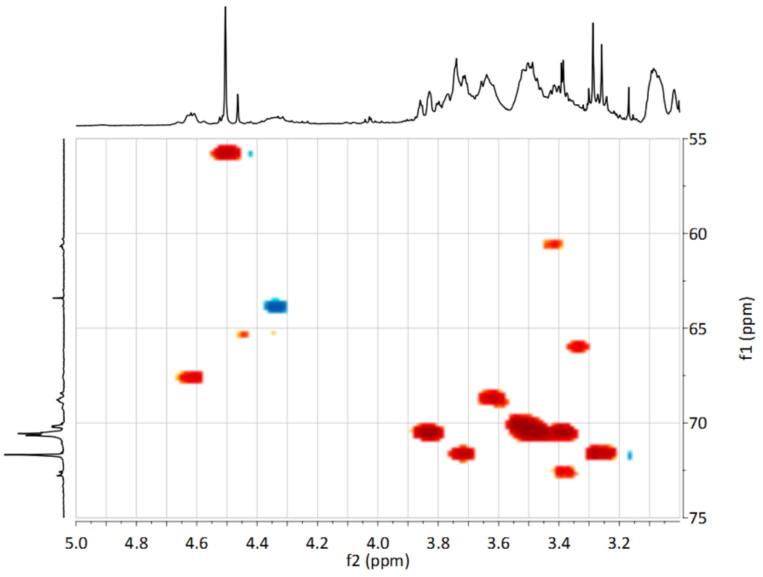

Figure S9. a) HSQC NMR spectra of HG40B5 at t = 0 h. b) Zoom of the region between 3.0 – 5.0 ppm in ^{1}H NMR and 55.0 – 75.0 ppm in ^{13}C NMR of the same HSQC NMR spectra. In the spectra, the blue signals correspond to -CH- and $-CH_3$ signals, while the red ones correspond to $-CH_2-$ signals.

a) HMBC spectra

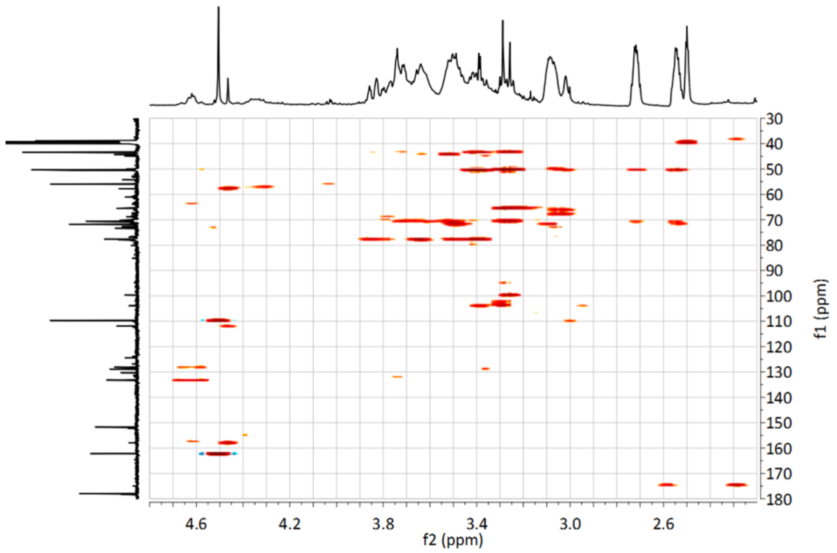

b) Zoom of HMBC spectra

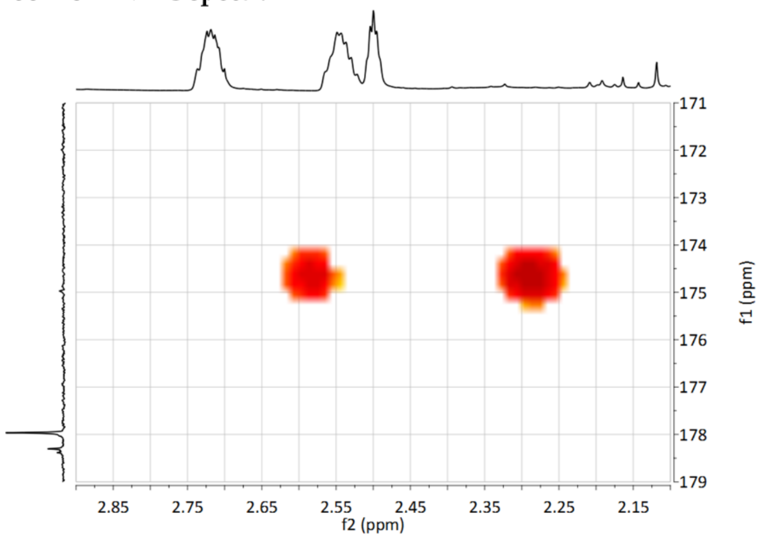

Figure S10. a) HMBC NMR spectra of HG40B5 at t = 0 h. b) Zoom between 2.10 - 2.90 ppm in ^{1}H NMR and between 171.0 – 179.0 ppm in ^{13}C NMR region of the same HMBC NMR spectra.

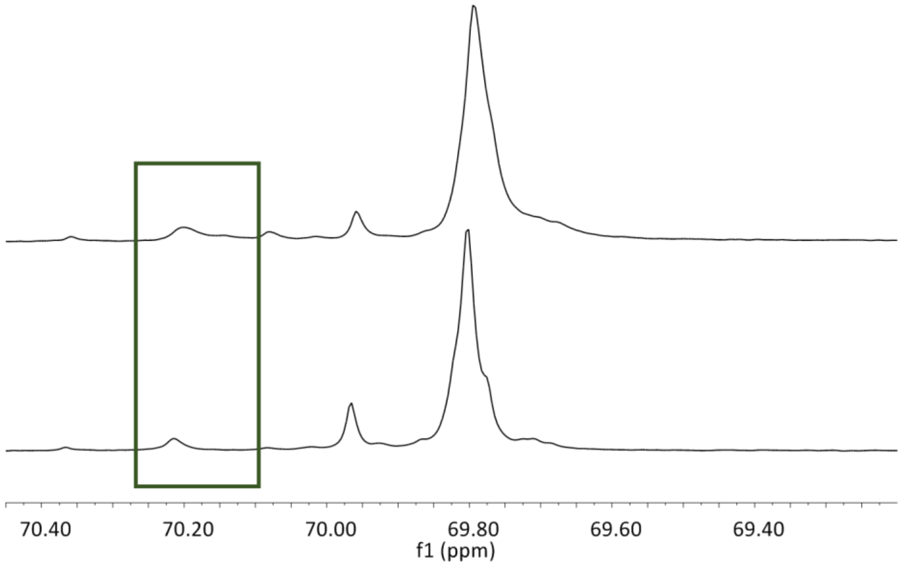

Figure S11. ^{13}C NMR spectra of HP40B5 at t = 0 h and t = 6 h.

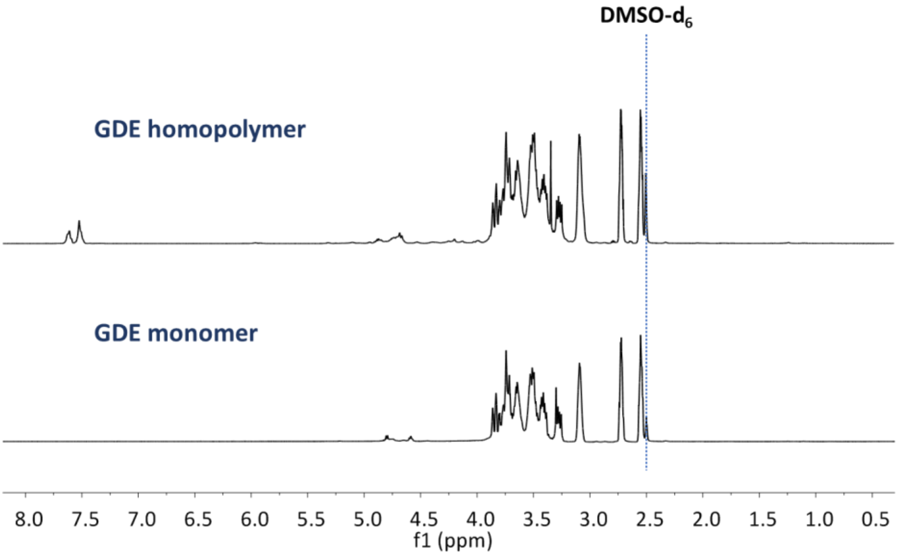

Figure S12. ^{1}H NMR spectra of GDE monomer and GDE homopolymer at t = 6 h.

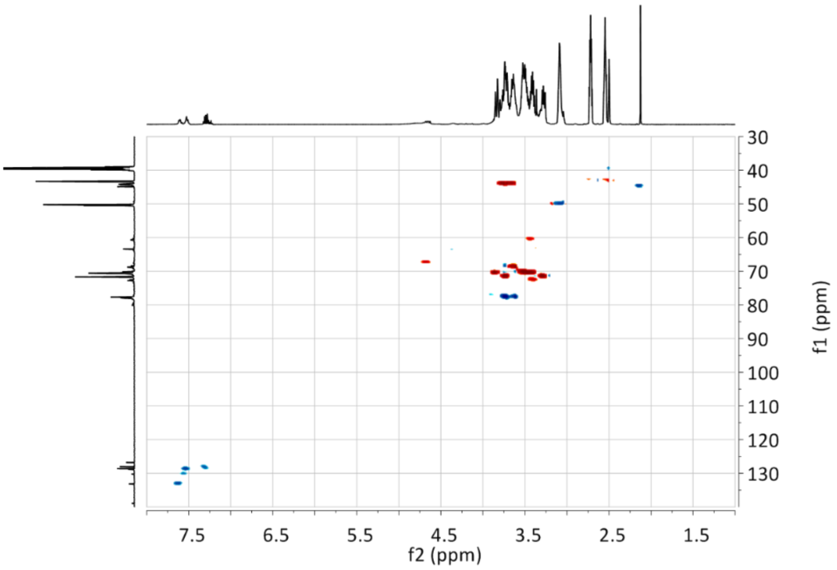

Figure S13. HSQC NMR spectra of GDE homopolymer at t = 0 h. In the spectra, the blue signals correspond to -CH- and $-CH_3$ signals, while the red ones correspond to $-CH_2-$ signals.

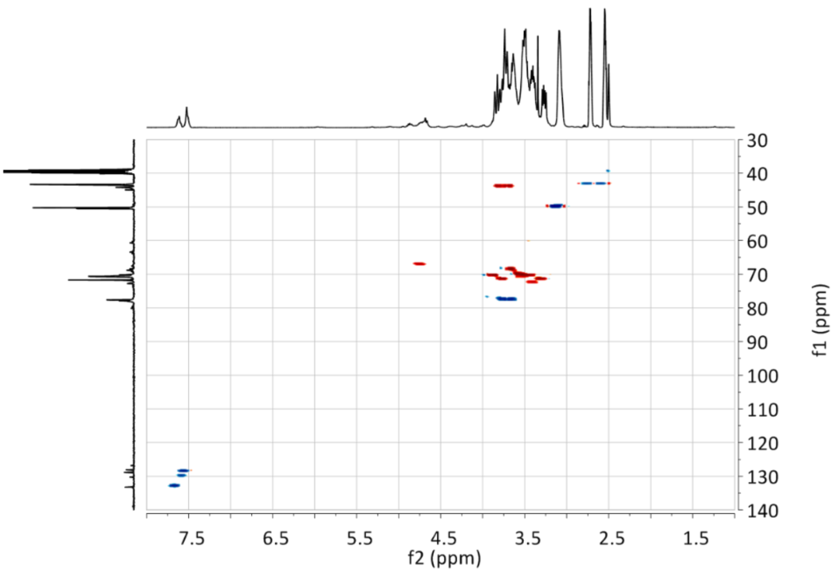

Figure S14. HSQC NMR spectra of GDE homopolymer at t = 6 h. In the spectra, the blue signals correspond to -CH- and $-CH_3$ signals, while the red ones correspond to $-CH_2-$ signals.

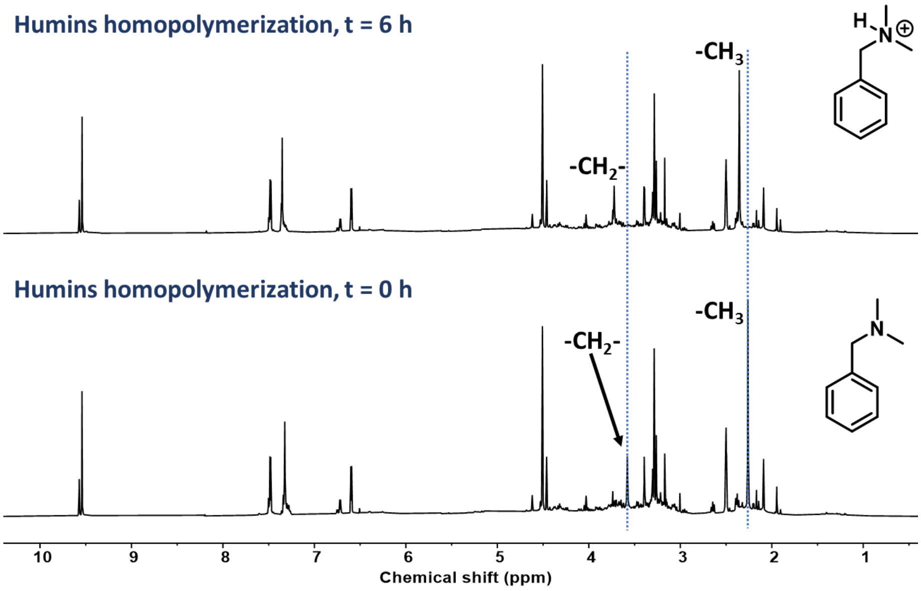

Figure S15. ^{1}H NMR spectra of humins with BDMA at t = 0 h and the same mixture at t = 6 h.

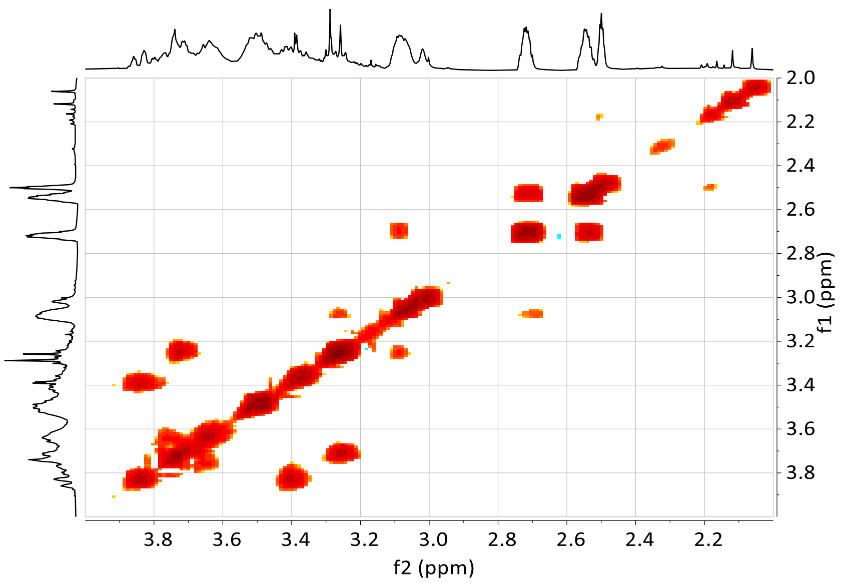

Figure S16. COSY NMR spectra of HG40B5 at t = 0 h.

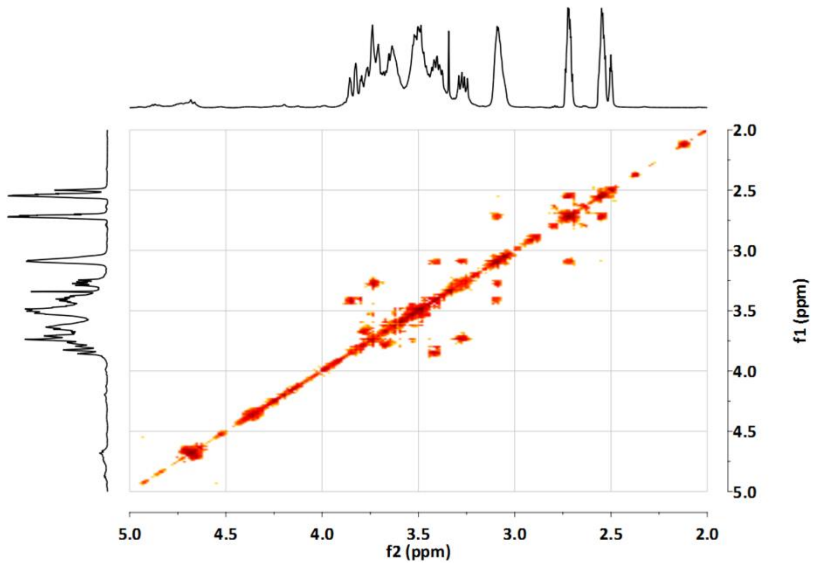

Figure S17. COSY NMR spectra of GDE95B5 at t = 6 h.

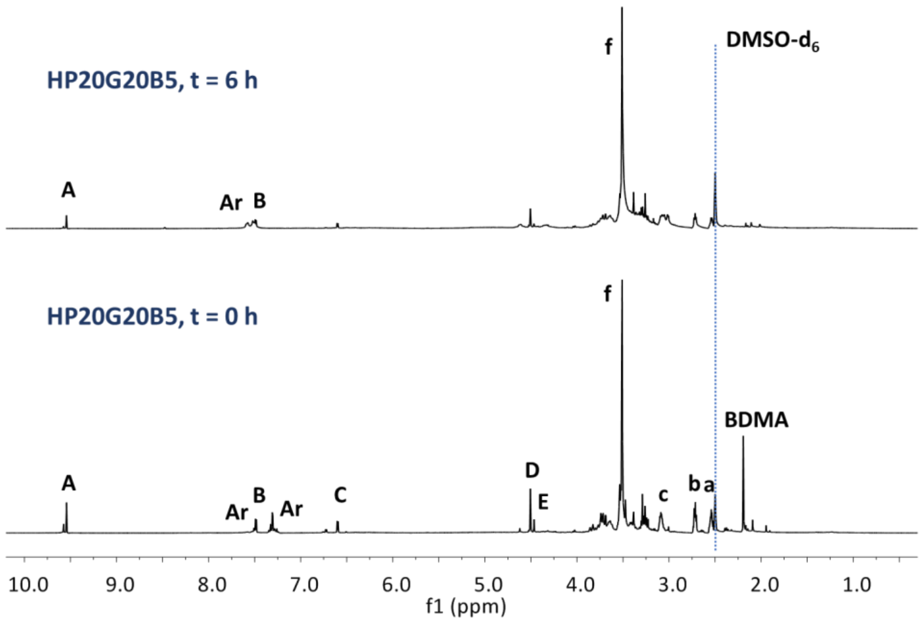

Figure S18. ^1H NMR spectra of HP20G20B5 at t = 0 h and at t = 6 h.

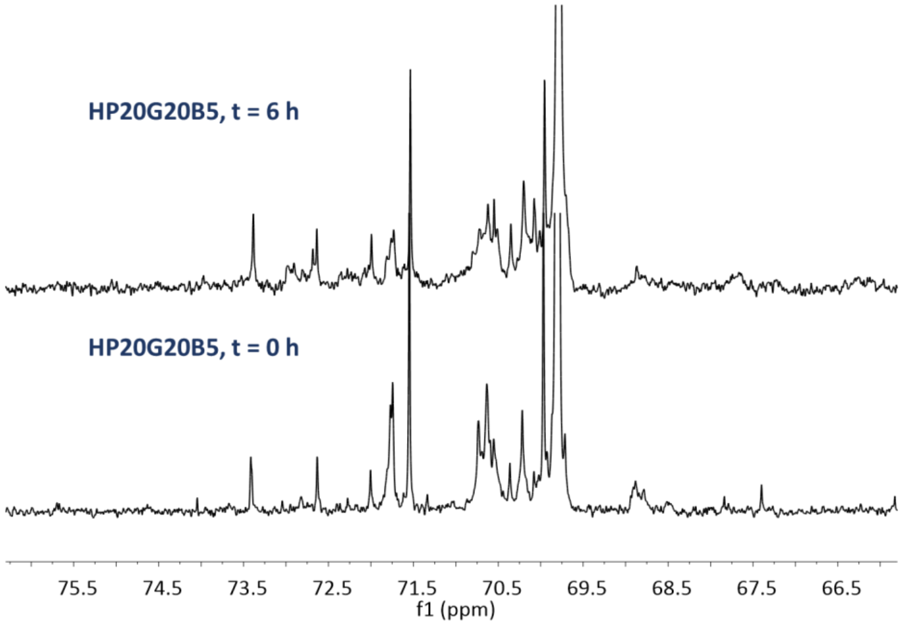

Figure S19. ^{13}C NMR spectra of HP20G20B5 at t = 0 h and t = 6 h.
